# Resin-acid derivatives as potent electrostatic openers of voltage-gated K channels and suppressors of neuronal excitability

**DOI:** 10.1038/srep13278

**Published:** 2015-08-24

**Authors:** Nina E Ottosson, Xiongyu Wu, Andreas Nolting, Urban Karlsson, Per-Eric Lund, Katinka Ruda, Stefan Svensson, Peter Konradsson, Fredrik Elinder

**Affiliations:** 1 Department of Clinical and Experimental Medicine, Linköping University, Linköping, Sweden; 2 Department of Physics, Chemistry and Biology, Linköping University, Linköping, Sweden

## Abstract

Voltage-gated ion channels generate cellular excitability, cause diseases when mutated, and act as drug targets in hyperexcitability diseases, such as epilepsy, cardiac arrhythmia and pain. Unfortunately, many patients do not satisfactorily respond to the present-day drugs. We found that the naturally occurring resin acid dehydroabietic acid (DHAA) is a potent opener of a voltage-gated K channel and thereby a potential suppressor of cellular excitability. DHAA acts via a non-traditional mechanism, by electrostatically activating the voltage-sensor domain, rather than directly targeting the ion-conducting pore domain. By systematic iterative modifications of DHAA we synthesized 71 derivatives and found 32 compounds more potent than DHAA. The most potent compound, Compound 77, is 240 times more efficient than DHAA in opening a K channel. This and other potent compounds reduced excitability in dorsal root ganglion neurons, suggesting that resin-acid derivatives can become the first members of a new family of drugs with the potential for treatment of hyperexcitability diseases.

Many diseases, that affect a large number of people, such as epilepsy, cardiac arrhythmia and chronic pain, depend on increased electrical excitability^1^. Unfortunately, not all of these diseases are entirely controlled by currently available pharmaceuticals. For instance, one third of the patients with epilepsy do not respond satisfactorily^2^,^3^,^4^ to existing treatment; therefore there is a need for new therapies. For all these conditions, voltage-gated ion channels, responsible for the generation and propagation of neuronal and cardiac action potentials, are obvious targets. These channels are tetrameric proteins with an ion-conducting pore at the center. Each of the four subunits has six transmembrane segments, named S1 to S6. The pore domain (S5-S6) includes the ion-conducting pore with the selectivity filter and the gates that open and close the pore^5^. The voltage-sensor domain (VSD, S1-S4) includes the positively charged voltage sensor S4 which moves through the channel protein during activation of the channel^6^,^7^,^8^.

Many clinically used drugs block voltage-gated ion channels by plugging the ion-conducting pore^2^,^9^ of Na, Ca, or K channels. Alternatively, instead of blocking the ion-conducting pore, a drug can act on either (i) the gate that opens and closes the channel, or (ii) the voltage sensor that controls the gate^10^ to affect the ion channel conductance. Retigabine, a new antiepileptic drug, opens the M-type K channel by acting on the gate and consequently shutting down electrical excitability^11^. Spider toxins and some other compounds specifically act on the VSD of the ion channel^12^,^13^,^14^ but there is no small-molecule pharmaceuticals targeting the VSD.

We have recently described a mechanism whereby polyunsaturated fatty acids (PUFAs) bind close to the VSD of different K channels and thereby electrostatically affect the charged voltage sensor in the VSD^15^,^16^,^17^,^18^. The hydrophobic and negatively charged PUFA molecule attracts the positively charged amino acid residues in the voltage sensor S4, thereby supporting an outward movement and rotation of S4 and consequently opening of the ion-conducting pore. The effect depends on (i) the number and geometry of the double bonds in the lipid tail^19^, and (ii) the charge of the PUFA molecule – the direction of the shift of the conductance curve along the voltage axis depends on the sign of the charge^16^. We refer to this as the lipoelectric mechanism^19^. The site of action for PUFAs is at the extracellular end of S3 and S4, distinct from previously described binding sites^15^, and it is mainly the final opening step of the channel that is affected^15^. Modifying the Shaker K channel by introducing two extra positively charged amino-acid residues in the extracellular end of the voltage sensor S4 (the 3R Shaker K channel) makes it highly sensitive to the PUFAs^17^. We took advantage of this increased sensitivity in screening for charged and lipophilic, i.e. lipoelectric compounds.

PUFAs have beneficial effects on epilepsy and cardiac arrhythmia^20^,^21^. However, relatively high concentrations of PUFAs are needed and the flexibility of the PUFA molecules makes them promiscuous to interact with other molecules and make them less likely to be developed into specific drugs; other types of drug-like small-molecule compounds are probably more suitable as drug candidates for treating hyperexcitability diseases. A possible starting point in the search for compounds acting via a lipoelectric mechanism is pimaric acid (PiMA (**1**); all compounds and nomenclature used in the paper are found in Supplementary Table S1). PiMA (**1**) opens the voltage-gated Shaker K channel (though to a lower extent than PUFAs)^17^, as well as the Ca-activated BK channel^22^. PiMA (**1**) belongs to the resin acids, a mixture of several related carboxylic acids found in tree resins, used to produce soaps for diverse applications^23^,^24^. Most of the resin acids belong to the diterpenoids, which are formed from four isoprene units, common organic molecules produced by many plants^25^. In the present investigation we report on the discovery of twelve compounds more potent than the PUFA docosahexaenoic acid (DHA), previously the most potent lipoelectric compound identified. We suggest that these compounds are good starting points to develop new drug candidates for the treatment of cardiac arrhythmia, epilepsy, and pain.

## Results

### Natural resin acids open the WT and the 3R Shaker K channel

**Figure 1:**
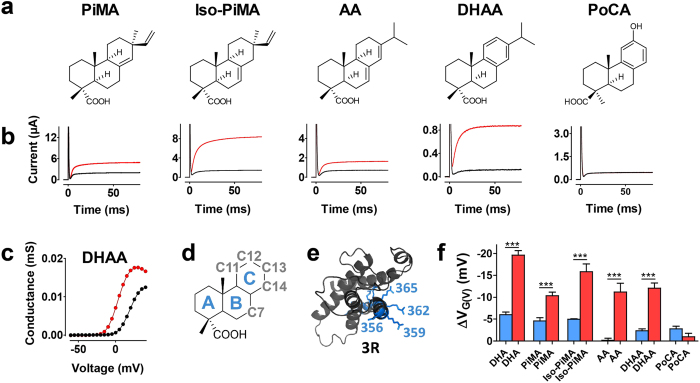
The effect of several natural resin acids on the opening of the Shaker K channel. (**a**) Molecular structure (from left to right) for pimaric acid (PiMA) (**1**), isopimaric acid (Iso-PiMA) (**2**), abietic acid (AA) (**3**), dehydroabietic acid (DHAA) (**4**), and podocarpic acid (PoCA) (**5**). (**b**) Representative current traces for voltages corresponding to 10% of maximum conductance in control solution at pH 7.4 of the 3R Shaker K channel. Black traces indicate control, and red traces 100 μM compound (same order as in a). (**c**) Representative *G(V)* curves for DHAA effects, same cell as in **b** (control, black symbols; DHAA, red symbols. ∆*V*_G(V)_ = −15.5. (**d**) Ring nomenclature of the general compound skeleton (blue) and modified carbons in derivates (grey). (**e**) Gating charge residues (362 = R1, and 365 = R2) and mutated residues (shown as R) of the 3R Shaker K channel (359R, and 356R) are marked on one VSD of the Shaker K channel in the active state. (**f**) Compound-induced *G(V)* shifts for the WT (blue) and 3R Shaker K channel (red). Mean ± SEM (n = 9, 15, 15, 6, 4, 4, 5, 5, 10, 6, 4, and 6 from left to right). Data for DHA (**6**) and PiMA (**1**) are from ref 17. The shifts of WT and 3R Shaker K channel are compared for each compound (one-way ANOVA together with Bonferroni’s multiple comparison test: *P < 0.05; ***P < 0.0001).

We investigated the effect of five naturally occurring and commercially available resin acids (Fig. 1a, pimaric acid, PiMA (**1**); isopimaric acid, Iso-PiMA (**2**); abietic acid, AA (**3**); dehydroabietic acid, DHAA (**4**); and podocarpic acid, PoCA (**5**)) at concentrations of 100 μM with pH 7.4 on the genetically modified 3R Shaker K channel. The channel was expressed in oocytes from *Xenopus laevis* and currents were measured by the two-electrode voltage-clamp technique (Fig. 1b). Four of the five resin acids had clear effects of the channel’s voltage sensitivity (Fig. 1c, Supplementary Table S1), but none was as efficacious as DHA (**6**), which has been used in previous studies on the lipoelectric mechanism^15^,^16^,^17^,^26^. Moving the double bond from the C ring (PiMA (**1**)) to the B ring (Iso-PiMA (**2**)) (Fig. 1d for nomenclature) increased the shift of the conductance-versus-voltage curve, *G*(*V*), from ΔV_*G*(*V*)_ = −10.4 ± 0.8 (*n* = 6) to −15.9 ± 1.8 mV (*n* = 4; *p* = 0.013). In contrast, the structural difference between AA (**3**) (conjugated double bond) and DHAA (**4**) (aromatic C ring) did not generate any difference in the *G*(*V*) shift (ΔV_*G*(*V*)_ = −11.2 ± 2 mV, *n* = 5, vs. −12.1 ± 1.7 mV, *n* = 6). In contrast, the fifth resin acid, PoCA (**5**), having a highly polar OH-group at the C-ring, had no effect on the *G*(*V*) for the 3R Shaker K channel (−1.4 ± 1.1; *n* = 5).

To explore if the investigated compounds acted via the lipoelectric mechanism we tested the resin acids on the wild-type (WT) Shaker K channel (where residues 359 and 356 are un-charged, Fig. 1e). PiMA (**1**), Iso-PiMA (**2**), AA (**3**), and DHAA (**4**), had much smaller effects on WT compared to the 3R Shaker K channel, suggesting they all act via the lipoelectric mechanism. However, AA (**3**) did not show any effects on the WT channel, suggesting that this compound probably will be difficult to turn into a potent lipoelectric compound on the WT channel. PoCA (**5**) on the other hand had no effect on the 3R or the WT Shaker K channel. The reason for the lack of effect of PoCA (**5**) was the OH-group in the C ring; exchanging this for hydrophobic groups increased the efficacy (**Compound 7–9**, Supplementary Fig. S1). However, none of these compounds produced the same extent of *G*(*V*) shift as the other resin acids. Further modification of the B-ring of PoCA did not increase the efficacy but reduced it (**Compound 10–12**, Supplementary Table S1). These initial experiments suggested that PiMA (**1**), Iso-PiMA (**2**) and DHAA (**4**) all were promising candidates to explore further. In the following experiments we focused on increasing the efficacy of DHAA (**4**).

### Side chains of the B-ring of DHAA (4) affect channel opening properties

**Figure 2:**
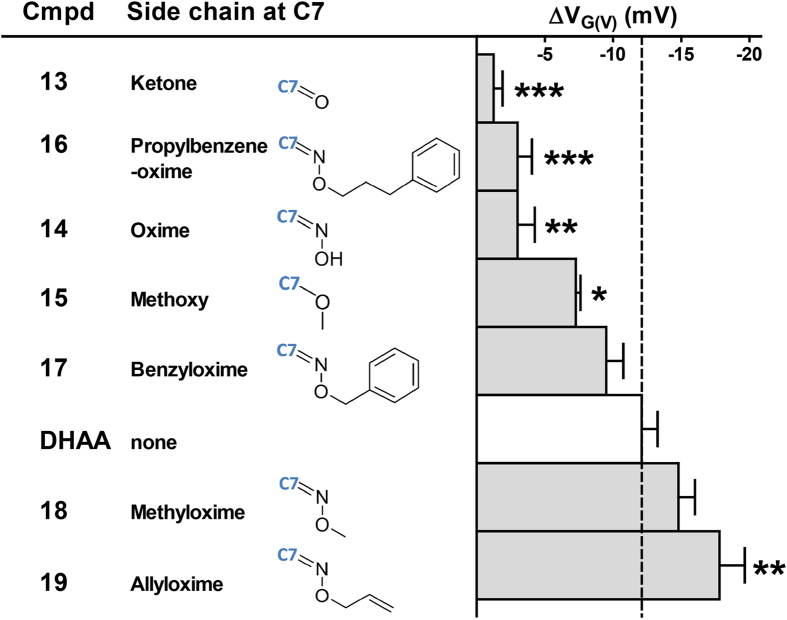
The effect of modifications of C7 at the B-ring for the activation of the 3R Shaker K channel. Chemical side chain and molecular structure for the introduced side chain at C7 of DHAA (**4**) (middle) for the specific compound (left). Compound(100 μM)-induced *G(V)* shifts for the 3R Shaker K channel (right). The dashed line equals the DHAA-induced shift. Mean ± SEM (n = 4–6). The shifts are compared with DHAA (**4**) (one-way ANOVA together with Dunnett’s multiple comparison test: *P < 0.05; **P < 0.01; ***P < 0.001).

As a first step, divergent substitutions were introduced in the B-ring of DHAA (**4**). We synthesized seven different side chains on C7 (**Compound 13–19**, Fig. 2, Supplementary Table S1). All polar substituents (**Compound 13–15**) clearly induced a smaller current increase by reducing the absolute *G(V)* shift. The polar properties probably makes it more difficult for the compound to integrate into the membrane. However, the introduction of the non-polar propylbenzen connected to the oxime group for **Compound 16** also caused a decreased efficacy compared to DHAA (**4**). This molecule is bulky which might complicate its integration into the membrane in close proximity to the voltage sensor. In support of this, shortening the chain length (**Compound 17**) restored the efficacy. The efficacies of two of the compounds (**Compound 17** and **18**) were not significantly different from to DHAA (**4**). One compound (**Compound 19**, an allyloxime on C7) significantly increased the effect compared to DHAA (**4**) (∆*V*_G(V)_ = −17.8 ± 1.9; *n* = 4). Taken together, these experiments indicate that the chemical properties of the B-ring might be important for interaction with the lipid bilayer or else have some steric effect for the interaction between the compound and the ion channel.

### Halogenation of C12 in combination with specific C7 side chains increases the efficacy to open K channel

In an attempt to increase the efficacy of the DHAA derivatives to open the 3R Shaker K channel by shifting its voltage dependence of activation, we introduced halogens to the C ring (Fig. 1d). Chlorination of C11 in DHAA clearly decreased the efficacy; halogenation (F, Cl, Br, or I) of C12 in DHAA had only minor effects; and fluorination of C14 in DHAA reduced while all other halogenations (Cl, Br, or I) of C14 increased the efficacy (Supplementary Fig. S2a). Double (C12 and C14) and triple (C11, C12 and C14) chlorinations decreased the efficacy, clearly showing that the effects of the chlorinations were not additive (Supplementary Fig. S2a).

**Figure 3:**
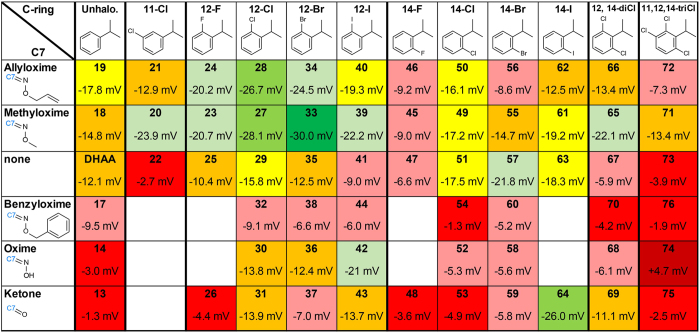
Halogenation-dependent potency. Shift of *G(V)* (mean values) by the unhalogenated and halogenated DHAA (**4**) derivates (100 μM), arranged according to halogenation of the C-ring (horizontal) and side chains attached to C7 (vertical). Compounds are color coded according to their potency: dark green: ∆*V*_G(V)_ ≤ −30.0 mV, green: −30.0 mV < ∆*V*_G(V)_ ≤ −25.0 mV, light green: −25.0 mV < ∆*V*_G(V)_ ≤ −20.0 mV, yellow: −20.0 mV < ∆*V*_G(V)_ ≤ −15.0 mV, orange: −15.0 mV < ∆*V*_G(V)_ ≤ −10.0 mV, light red: −10.0 mV < ∆*V*_G(V)_ ≤ −5 mV, red: −5.0 mV < ∆*V*_G(V)_ ≤ 0.0 mV, dark red: ∆*V*_G(V)_ > +0.0 mV.

To further explore this non-additivity, we tested combinations of C7 side chains and C-ring halogenations. For instance, bromination of C12 with a methyloxime at C7 increased the *G*(*V*) shift, in absolute terms, from −14.8 to −30.0 mV, while bromination of C12 in the absence of a side chain at C7 had no effect (Fig. 3). In total we explored 71 combinations of C7 side chains with halogenations to C11 (**Compounds 20–22**), C12 (**Compounds 23–44**), C14 (**Compounds 45–64**), or combinations of C11, C12, and C14 (**Compounds 65–76**). Their capacity to shift the voltage-dependence of activation, the *G*(*V*) curve, of the 3R Shaker K channel was measured at 100 μM with pH 7.4 (Fig. 3, Supplementary Table S1). The results of the combinations suggest five main findings: (i) halogenation of C12 increased the effect except if C7 lacked a side chain or was very bulky (benzyloxime), (ii) halogenation of C14 had modest effects with a few exceptions, (iii) fluorination was the least effective halogenation, (iv) iodination increased the effect in combination with short polar side chains at C7, and (v) double and triple chlorination reduced the effect compared to chlorination of C12 alone (Fig. 3). The five most effective combinations contained either a chlorine or a bromine at C12 together with either a methyloxime or an allyloxime at C7 (**Compounds 27–28, and 33–34**), or an iodine at C14 in combination with a ketone at C7 (**Compound 64**). The ΔV_*G*(*V*)_ were between −24.5 and −30.0 mV, compared to −12.1 mV for DHAA (**4**).

These results show that the *G*(*V*)-shifting properties introduced by halogenations in the C ring and by different side chains at C7 are not additive. The effect of this non-additivity can be quantified by Δ*V*_tot_ = Δ*V*_halo_ + Δ*V*_C7_ + Δ*V*_int_, where Δ*V*_tot_ is the total difference in *G*(*V*) shift compared with DHAA, Δ*V*_halo_ is difference in *G*(*V*) shift compared with DHAA if only the halogenation is introduced, Δ*V*_C7_ is difference in *G*(*V*) shift compared with DHAA if only side chain modifications at C7 is introduced, and Δ*V*_int_ is the remaining interaction component – the deviation between the expected additive value and the measured value. Δ*V*_int_ follows a characteristic pattern for each halogenation: (i) Halogenation of C11 and C12 in combinations with C7 modifications had a large positive cooperative effect (i.e. increased negative shift) while halogenation of C14 in combination with modifications of C7 had a large negative cooperative effect (i.e. decreased negative shift). (ii) The most hydrophilic side chains (ketone, oxime and methyloxime) had a slightly larger positive cooperative effect than the most hydrophobic side chains (allyloxime and benzyloxime) (Supplementary Fig. S2c).

Thus, to summarize, neither halogenation of C12, nor a side chain modifications at C7 had a large effect *per se*, but the combination of either methyloxime or allyloxime at C7 and halogenation at C12 had a large effect. In contrast, halogenation (except F) of C14 had a slightly larger effect, but in combination with the different side chains at C7 this effect was largely suppressed.

### Replacement of the C13 isopropyl with a chloride increase the efficacy to open a K channel

**Figure 4:**
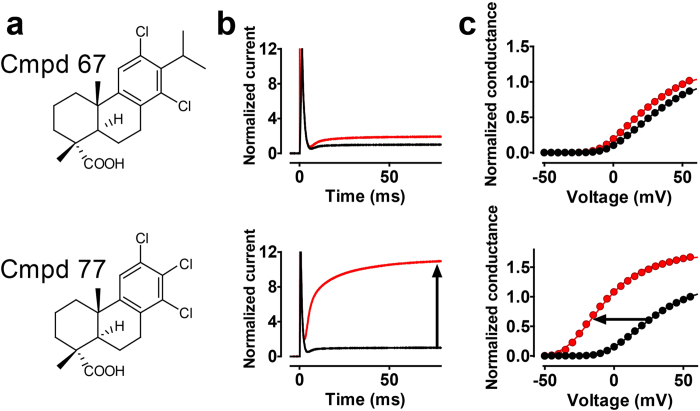
Replacement of the isopropyl group at C13 with a chlorine. (**a**) Molecular structure for **Compound 67** (upper) and **Compound 77** (lower). (**b**) Representative current traces for voltages corresponding to 10% of maximum conductance in control solution at pH 7.4 of the 3R Shaker K channel. Black traces indicate control, and red traces 100 μM compound (upper, **Compound 67**; lower, **Compound 77**). (**c**) Representative *G(V)* curves. Same cells as in **b** (control, black symbols; compound, red symbols. ∆*V*_G(V)_ = −6.1 mV by **Compound 67** (upper), and −32.6 mV by **Compound 77** (lower) in these examples.

In addition to the DHAA-derivatives described above, a compound (**Compound 77**) similar to **Compound 67** but with the isopropyl of the C-ring replaced by a chloride (Fig. 4a) was synthesized and tested on the 3R Shaker K channel. The efficacy of this compound was, in contrast to **Compound 67**, very high. For the 3R Shaker K channel, 100 μM **Compound 77** at pH 7.4 caused more than a ten-fold increase of the current compared to less than a twofold increase for **Compound 67** (Fig. 4b) and also a five times larger shift of the voltage dependence of activation (−31.6 ± 2.1 mV, *n* = 9, and −5.9 ± 0.4 mV, *n* = 5 for **Compound 77** and **Compound 67**, respectively; Fig. 4c).

### Increased channel-opening propensity depends on decreased apparent p*K*_a_ value, increased potency, and increased shift of the deprotonated compound

We have identified twelve compounds that are more efficacious than DHA, the most potent *G*(*V*) shifter described so far^17^,^19^, and 31 compounds more efficacious than DHAA, which was the starting molecule for this synthetic work (Supplementary Table S1). To explore if the derivatives act via the lipoelectric mechanism, as has been described for DHA (**6**) and other PUFAs^17^,^19^, and to explore if the difference in efficacy among the derivatives depends on altered p*K*_a_ values of the compound, altered potencies, or on altered maximum shifts we tested the effects at different concentrations, pH values, and channels (WT vs. 3R). We limited these tests to the starting molecule, DHAA (**4**), and the two most potent derivatives, **Compound 33** and **77**.

**Figure 5:**
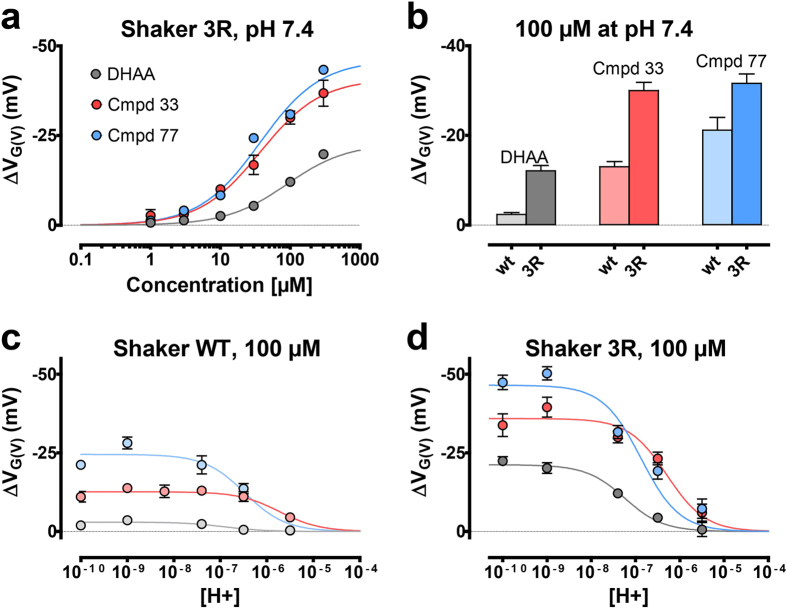
Concentration and pH dependent compound effects. (**a**) Concentration response curve for DHAA (**4**) (grey), **Compound 33** (red), and **Compound 77** (blue) at pH 7.4 for the 3R Shaker K channel. Error bars indicate SEM (n = 4–9). (**b**) Compound-induced *G(V)* shifts for the WT and 3R Shaker K channels. (**c,d**) pH dependence curves for WT (**c**) and 3R Shaker K channel (**d**) for 100 μM DHAA (**4**) (grey), 100 μM **Compound 33** (red), and 100 μM **Compound 77** (blue). Error bars indicate SEM (n = 4–9).

**Compound 77** induced a significant shift at 1 μM at pH 7.4 on the 3R Shaker K channel (∆*V*_G(V)_ = −1.3 ± 0.3 mV; *n* = 9) (Fig. 5a). The EC_50_-values were 89 μM for DHAA (**4**), and 37 μM for both derivatives. The maximum shifts were −23 mV for DHAA (**4**), and −41 mV and −46 mV for **Compound 33** and **77** respectively. Thus, both the potency and the maximum shift were increased in the derivatives. For all three compounds, the induced shifts are smaller on Shaker WT compared to Shaker 3R K channels (Fig. 5b), thus supporting a lipoelectric mechanism. Is the increased potency and increased maximum shift at pH 7.4 in part caused by alterations in the p*K*_a_ value of the compounds? In previous studies, we have shown that the effects of both DHA (**6**)^15^,^16^,^19^ and PiMA (**1**)^17^ depend on pH, with an increased potency at higher pH. This is explained by the incomplete deprotonation of the compounds at pH 7.4 due to a low local pH close to the membrane. (The apparent p*K*_a_ value of PUFAs in a lipid membrane is about 7.5 in Shaker WT)^19^. At higher pH the compound is expected to be fully deprotonated and thus completely negatively charged producing a larger effect on the positively charged voltage sensor than at neutral pH. The apparent p*K*_a_ values for DHAA (**4**) were 7.3 and 7.2 for the 3R and WT channels respectively (Fig. 5c,d), the apparent p*K*_a_ values for **Compound 33** were 5.8 and 6.1 respectively, and the apparent p*K*_a_ values for **Compound 77** were 6.5 and 6.8 respectively (Fig. 5c,d). Thus the derivatives but not the starting molecule DHAA (**4**) are almost fully deprotonated at neutral pH. Taken together, modifications increasing the *G*(*V*) shift had profound effect on the apparent p*K*_a_ value (up to 1.5 pH steps), the potency and the maximum shift. All this makes **Compound 77** a much better shifter than DHAA (**4**); 100 μM at pH 7.4 induces a tenfold larger *G*(*V*) shift for WT channels, −21.2 mV for **Compound 77** vs. −2.3 mV for DHAA.

### The resin-acid derivatives are more potent in a mammalian expression system

The *Xenopus* oocyte as an expression system can affect the absolute compound concentration required to reach a certain effect compared to mammalian cells^27^. To test if the compound effects reported in the present study also apply to K channels expressed in mammalian cells, we investigated the effects of selected compounds (**Compounds 13**, **28**, **29**, **33**, **55**, and **77**) on a Chinese hamster ovary (CHO) cell-line stably expressing the 3R Shaker K channel using whole-cell patch-clamp recordings. DHA (**6**) was about 7 times more potent on channels expressed in CHO cells than in *Xenopus* oocytes (Supplementary Fig. S3a,b). Shifts similar to those observed in oocytes (at 100 μM concentration) were observed in CHO cells using lower (10 μM) concentrations of resin-acid derivates (Supplementary Fig. S3c). Thus, the described *G(V)* shifting property of these compounds is a general effect coupled to the specific ion channel and not to the expression system.

To test all compounds that induced a large shift in the *G(V)* of channels expressed in *Xenopus* oocytes on channels expressed in the CHO cell line, and to cover a large concentration range, we performed experiments using the IonWorks Quattro automated patch-clamp system (Molecular Devices). Eight concentrations (from 11 nM to 33 μM) of the 20 most potent compounds found in the oocyte experiments were tested at four membrane voltages (0, 20, 40, and 60 mV). The effect, measured as a relative ion current increase, was largest at 0 mV and smallest at +60 mV as expected for a negative shift along the voltage axis of the *G(V)* curve. For quantitative evaluations we used data from +20 mV. For **Compound 77**, 3.3 μM significantly increased the current, and 33 μM increased the current 8-fold (Supplementary Fig. S4a). Nine compounds showed a significant effect at 3.3 μM (**Compounds 23, 27, 28, 33, 34, 39, 40, 65, 77**). There was a clear correlation between data from the CHO cell line measured by IonWorks and data from the *Xenopus* oocytes measured by the two-electrode technique (Supplementary Fig. S4b).

Taken together, the lipoelectric compounds synthesized in the present investigation act on K channels expressed in a mammalian cell line, and nine of the compounds significantly increase the channel opening at 3.3 μM, suggesting that these compounds could potentially be developed into excitability-reducing compounds. To investigate this possibility, we studied if these compounds reduced excitability in neurons with endogenously expressed K channels.

### Resin-acid derivatives hyperpolarize DRG neurons and reduce neuronal excitability

**Figure 6:**
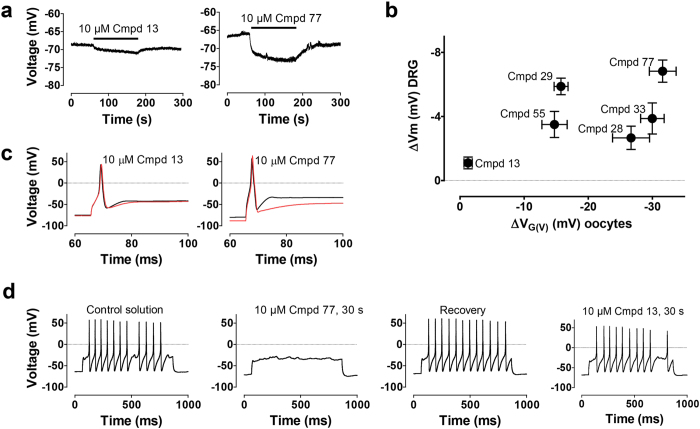
The effect of compounds on the resting membrane potential (V_m_) and excitability of DRG neurons. (**a**) Recordings of V_m_ during application of **Compound 13** and **77**. (**b**) Compound-induced hyperpolarizing shifts of V_m_ of DRGs (10 μM at pH 7.4) plotted versus the shifts for the 3R Shaker K channel expressed in *Xenopus* oocytes (100 μM at pH 7.4). Mean ± SEM (for DRG-recordings, n = 4–7; for oocyte recordings, n = 4–9). (**c**) Effects on single action potentials by **Compound 13** and **77** (black is control and red is test compound). (**d**) Effects on repetitive firing elicited by a continuous current pulse. The effect of **Compound 77** is clearly reversible. **Compound 13** had almost no effect.

Selected compounds (**Compounds 13**, **28**, **29**, **33**, **55**, and **77)** were also tested on native dorsal root ganglion (DRG) neurons from mice. All of the tested compounds caused significant shifts of the resting membrane potential (∆*V*_m_) towards more negative voltages (p < 0.05; Fig. 6a,b). This hyperpolarization most likely depends on the opening of one or several types of K channels. The excitability of these neurons is expected to be reduced by increasing the amount of the input required to reach action potential threshold. **Compound 13**, the least potent *G(V)* shifter of the investigated compounds, caused only a small alteration of the resting potential (∆*V*_m_ = −1.1 ± 0.4 mV; *n* = 5), and **Compound 77**, the most potent shifter, caused the largest hyperpolarization (∆*V*_m_ = −6.8 ± 0.7 mV; *n* = 6) (Fig. 6a). The relationship between the *G(V)* shift in *Xenopus* oocytes and the hyperpolarization in DRG neurons is clear, but there is a clear scatter in the data (Fig. 6b). A reason for this scatter might be that the channel(s) present in the DRG neurons is different from the 3R Shaker K channel^28^. The two most potent compounds on the DRG neurons (**Compound 29** and **77**) both lack a C7 side chain combined with the presence of a chlorine at C12. The compounds causing intermediate hyperpolarization (**Compound 28**, **33**, **55**) have side chains on C7. Thus, the presence of a side chain on C7 may be less important for hyperpolarizing the membrane potential in DRG neurons.

To test if the compounds also reduced neuronal excitability, we stimulated the DRG neurons by a constant current pulse. Two types of neurons were defined by whether they responded to this current pulse with (a) a single action potential or (b) a train of action potentials. We then evaluated the effects of a poor shifter (**Compound 13**) and a strong shifter (**Compound 77**), as defined by the oocyte and CHO cell experiments. In type a neurons, 10 μM of **Compound 13** had almost no effect the resting membrane potential (Fig. 6c **left**), no effect on minimum current needed to elicit an action potential (+1 ± 8%, p = 0.88, *n* = 6), and only a very small effect on the afterhyperpolarization (Fig. 6c **left**). In contrast, 10 μM of **Compound 77** made the resting membrane potential more negative (Fig. 6c **right**), increased the minimum current needed to elicit an action potential (+11 ± 4%, p < 0.05, *n* = 6), and had a pronounced effect on the afterhyperpolarization (Fig. 6c **right**), suggesting that a K channel stayed open for a prolonged time after the action potential likely reducing excitability. All these effects are consistent with actions only on Kv channels. In type b neurons, (Fig. 6d **panel 1**) 10 μM of **Compound 77** completely abolished all action potentials (**panel 2**), or all but the initial action potential (*n* = 3). The effect was clearly reversible (**panel 3**). In contrast, 10 μM of **Compound 13**, had almost no effect (**panel 4**). Thus, compounds displaying *G*(*V*)-shifting properties in *Xenopus* oocytes are also able to reduce excitability in neurons.

## Discussion

We have described the systematic design, synthesis and functional characterization of several resin-acid derivatives with potential anti-epileptic and/or analgesic properties. Halogenation of C12 and small non-polar side chains on C7 increased the capacity of resin-acid derivates for opening a K channels expressed in two different cellular systems. The channel-opening properties of the compounds were also correlated with the ability to reduce excitability in DRG neurons. Thus, the described resin-acid derivatives can be interesting lead compounds in the development of medications aimed at reducing neuronal excitability in conditions such as epilepsy and pain.

**Compound 77**, the most potent compound described in the present investigation, shifts the *G*(*V*) of WT Shaker K channels at 100 μM with pH 7.4 by −21.2 mV, a considerable improvement compared to the −2.3 mV shift produced by the starting molecule DHAA (**4**) and −6.0 mV shift for DHA (**6**)^17^,^19^, the most potent lipoelectric compound prior to this study. At the relatively negative voltages critical for repetitive firing in neurons^20^ these shifts can be converted to increases in current amplitude, *A* = exp(−Δ*V*/4.7)^16^. In the WT Shaker K channel **Compound 77** thus increases the current by a factor of 25 compared with DHA (**6**), and by a factor of 56 compared with DHAA **(4)** (only differing in having three chlorines instead of an isopropyl group in the C ring). This gain factor (between **Compound 77** and DHAA) increases to 240 in the 3R Shaker K channel. Combining a number of modifications makes this gain immense; if 100 μM DHAA on the WT channel at pH 7.4 is compared with 100 μM of an isopropyl-lacking chlorided derivative (**Compound 77**) on an arginine-enriched channel (3R Shaker K channel) in a proton-depleted solution (pH 9.0), then the gain in current is 44,000.

Some of the compounds described in the present investigation were previously reported to affect the current through large-conductance Ca-activated K (i.e. BK) channels^29^,^30^,^31^. However, for the BK channel it has been suggested that the intracellular linker between S6 and the cytosolic Ca binding site is the determinant for this effect^32^. It has also been reported that the effect of polyunsaturated fatty acids on BK channels depends on the co-expressed β-subunit^33^,^34^. In addition, dissimilar effects have been found for the BK channel and the 3R Shaker channel (Supplementary Fig. S5) suggesting that the mechanisms and sites of actions might be different.

The present study has focused on the Kv1-type Shaker K channel and a modification of this channel (the 3R channel). So far there are no reports of resin acids and their derivatives on other channels than the Shaker channel or the BK channel. However, given the similarity in effects between the DHAA derivatives and the PUFAs, it is possible that also other ion channels are affected by the DHAA derivatives investigated in the present study. Simultaneous action on several types of channels can even be advantageous when designing drugs against epilepsy^35^. By using a scaling factor of 7 to convert potency between mammalian cells and the *Xenopus* oocytes (Supplementary Fig. S5b), we estimate that 14 μM of **Compound 77** shifts the *G*(*V*) by −21 mV at pH 7.4 in mammalian cells, and that 14 μM of DHAA (**4**) shifts the *G*(*V*) by −2.3 mV. In a computational study by Tigerholm *et al* (2012)^35^ it was found that shifts between −1 and −6 mV for several types of channels simultaneously efficiently reduce hyperexcitability in CA1 hippocampal neurons. While at least 6 μM DHAA (**4**) is needed to cause shifts within this range of the WT Shaker K channels investigated in the present study, less than 0.6 μM **Compound 77** is needed to cause the same shift. Thus, small modifications of the chemical structure of resin acids dramatically can increase their efficacy in the sub-micromolar concentration range, making them potential starting points for the development of new medications for indications such as epilepsy and pain.

## Methods

### Shaker K channels

All animal experiments were approved by the Linköping’s local Animal Care and Use Committee and the experiments were performed in accordance with relevant guidelines and regulations. A modified Shaker H4 channel^36^, with removed N-type inactivation (ShH4IR)^37^, here called the wild-type (WT) channel, was expressed in oocytes. In addition, a modified 3R Shaker K channel, where two introduced positive charged arginines (M356R and A359R) in addition to one native arginine (R362) makes the channel more sensitive to DHA^17^, was expressed in oocytes and in a CHO-K1 stable cell line.

### Preparation and injection of oocytes

African clawed frogs (*Xenopus laevis)* were anesthetized with 1.4 g/L ethyl 3-aminobenzoate methanesulfonate salt (tricaine). After an incision through the abdomen a batch of oocytes was removed. Clusters of oocytes were separated by incubation for ~1 h in a Ca-free O-R2 solution (in mM: 82.5 NaCl, 2 KCl, 5 HEPES, and 1 MgCl_2_; pH adjusted to 7.4 by NaOH) containing Liberase Blendzyme. The oocytes were then incubated at 8 °C in a modified Barth’s solution (MBS; in mM: 88 NaCl, 1 KCl, 2.4 NaHCO_3_, 15 HEPES, 0.33 Ca(NO_3_)_2_, 0.41 CaCl_2_, and 0.82 MgSO_4_; pH adjusted to 7.6 by NaOH) supplemented with penicillin (25 U/ml), streptomycin (25 μg/ml), and sodium pyruvate (2.5 mM) for 2–24 hours before injection. 50 nl of cRNA (50 pg) were injected into each oocyte using a Nanoject injector (Drummond Scientific, Broomall, PA). Injected oocytes were kept at 8 °C in MBS until one day before electrophysiological recordings, after which they were incubated at 16 °C. All chemicals were supplied by Sigma-Aldrich (Stockholm, Sweden) unless stated otherwise.

### Generation and maintenance of CHO-K1 cell lines stably expressing 3R channels

A stable CHO-K1 cell line expressing the 3R Shaker K channel was generated as follows. The construct was cloned into the pcDNA3 vector. CHO-K1 cells were plated into T75 culture flasks and grown without penicillin/streptomycin. After 24 h, cells were transfected with 25 μg of expression construct and of 60 μl Lipofectamin 2000 according to the manufacturer’s protocol. For polyclonal selection, cells were grown in the presence of G418 (400 μg/ml). Single cells from G418 resistant cell pools were plated into 96 well plates for dilution cloning. Monoclonal cell lines from single colonies were expanded and tested electrophysiologically for ion channel expression. One stable monoclonal CHO-K1 cell line expressing the 3R Shaker K channel was selected and used for testing all compounds. For manual electrophysiology, cells were cultured in F-12 Nutrient Mixture (Ham) with GlutaMAX™ supplemented with 10% fetal calf serum, penicillin/streptomycin and G418 (200 μg/ml) at 37 °C with 5% CO_2_. For automated electrophysiology, cells were cultured in DMEM/F12+ Glutamax (Gibco), supplemented with 10% fetal calf serum, 1% non-essential amino acids (Invitrogen), and G418 (400 μg/ml) at 37 °C with 5% CO_2_.

### Preparation of dissociated neurons from dorsal root ganglia

All animal experiments were approved by the local Animal Care and Use Committee and followed international guidelines. Eight female 7–12 week-old C57BL/6 mice (Scanbur) were used for this study. The mice were anaesthetized with isoflurane and decapitated. Dorsal root ganglia (DRG) were dissected from all spinal levels and enzymatically digested with collagenase (250 CDU/ml) for 15 min, and then trypsin (1 mg/ml) was added for another 30 min. Ganglia were centrifuged and resuspended in Leibovitz L-15 media with glutamine, supplemented with 10% fetal calf serum, 38 mM glucose, 24 mM NaHCO3 and penicillin/streptomycin. Ganglia were triturated and the DRG neurons plated onto poly-D-lysine coated plastic coverslips. Neurons were cultured at 37 °C with 5% CO_2_ for 1–3 days before the electrophysiological recordings.

### Manual electrophysiology

All manual electrophysiological recordings were performed at room temperature (20–23 °C), using a GeneClamp 500B amplifier (for oocytes) or a Axopatch 200B amplifier (for CHO-K1 cells and DRG neurons) and a Digidata 1440A digitizer and pClamp 10 software (all from Molecular Devices, Inc., Sunnyvale, CA, USA). Compounds were initially dissolved at 100 mM in 99.5% EtOH and stored at −20 °C. Compounds were subsequently diluted to the desired test concentration in extracellular solution.

#### Manual two-electrode voltage-clamp (TEVC) of oocytes

The oocyte was placed in a bath surrounded by 1K extracellular solution that contained (in mM): 88 NaCl, 1 KCl, 15 HEPES, 0.4 CaCl_2_, and 0.8 MgCl_2_, pH adjusted to 7.4 by NaOH (reaching a sodium concentration of ~100 mM). Control solution was added to the bath with a gravity driven perfusion system. Compound solution was added to the bath manually with a syringe. Two glass microelectrodes were inserted into the oocyte using micromanipulators. The microelectrodes were pulled from borosilicate glass, filled with 3M KCl and had a resistance of 0.5–2 MΩ. All channels were closed when the membrane potential was clamped to −80 mV and this voltage was set as the holding potential. Currents were evoked from the holding potential of −80 mV by 100-ms long, 5-mV steps ranging from −80 up to +50 mV (WT) or +70 mV (3R).

#### Manual whole-cell patch clamp of CHO-K1 cells and DRG neurons

CHO-K1 cells were plated on coverslips 2 h before the manual electrophysiological recordings. Coverslips with CHO-K1 cells or DRG neurons were placed in a recording chamber and perfused with extracellular solution by a gravity-fed perfusion system. Compounds were applied by a pressurized, automated OctaFlow perfusion system (ALA Scientific Instruments). The signals were sampled at 5–20 kHz after low-pass filtering at 2–5 kHz. For CHO-K1 cells the intracellular solution contained (in mM): 120 K-gluconate, 10 KCl, 5 EGTA, 10 HEPES, 4 Mg-ATP, 0.3 Na-GTP, pH 7.3; the extracellular solution contained (in mM): 135 NaCl, 4 KCl, 10 HEPES, 1 MgCl_2_, 1.8 CaCl_2_, 10 glucose, pH 7.4. Currents were evoked in CHO-K1 cells in whole-cell voltage-clamp mode from a holding potential of −80 mV by 100-ms long, 10-mV steps ranging from −80 to +80 mV.

For DRG neurons the intracellular solution contained (in mM): 120 K-gluconate, 10 KCl, 1 EGTA, 10 HEPES, 4 Mg-ATP, 0.3 Na-GTP, pH 7.3; the extracellular solution contained (in mM): 144 NaCl, 2.5 KCl, 10 HEPES, 0.5 MgCl_2_, 2 CaCl_2_, 10 glucose, pH 7.4. A liquid-junction potential of approximately −14 mV was corrected for. Pipettes were pulled from borosilicate glass with a vertical patch electrode puller PIP5 (HEKA) and had a resistance of 4–6 MΩ when filled with intracellular solution. Small and medium sized DRG neurons were selected for whole-cell current-clamp recording. 10 μM of the compounds were applied for 120 s to study the effect on the resting membrane potential, and for 30–120 s to study the effects on evoked action potentials. Action potentials were evoked by 400–800-ms depolarizing pulses from 0.03 up to 2 nA in steps of 0.03 to 0.2 nA. Only neurons with a resting membrane potential more negative than −60 mV were used in this study.

### Automated planar patch-clamp electrophysiology

Recordings were performed in CHO-K1 cells using the PPC mode of the IonWorks Quattro automated patch clamp system (Molecular Devices, Inc., Sunnyvale, CA, USA). The operation protocol for this system has been previously described^38^,^39^. The intracellular solution contained (in mM): 100 K-glutamate, 40 KCl, 3.2 MgCl_2_, 3 EGTA and 5 HEPES (pH 7.25–7.3 with KOH) and the extracellular solution was D-PBS, (Gibco) and contained (in mM): 138 NaCl, 2.7 KCl, 1.5 KH_2_PO_4_, 8 Na_2_HPO_4_, 0.9 CaCl_2_, 0.5 MgCl_2_ and 5.5 glucose. To establish the whole-cell perforated patch configuration, 25 mg/ml amphotericin B was added to the intracellular solution, giving a final concentration of 150 μg/ml. Wells with a series resistance of ≤20 MΩ or with a current <0.05 nA were not included in the analysis. The current signal was sampled at 10 kHz.

All compounds were dissolved in 100% dimethylsulfoxide (DMSO) to a final stock concentration of 10 mM. Half log serial dilutions were prepared in DMSO, where the final DMSO concentration in the external recording solution was 0.3%.

Prior to electrophysiological recordings, the cells were incubated at 30 °C with 5% CO_2_ during the last 15−24 h, washed with Mg and Ca-free PBS, detached with tryplee, centrifuged and resuspended in D−PBS, and finally added to the IonWorks cell boat.

Briefly, whole-cell perforated patch recordings were made at room temperature (∼21 °C). After attaining the whole-cell configuration, the voltage protocol was applied once to obtain a control recording (pre-scan) and again after compound application (post-scan) to measure potentiation of potassium currents. Cells were incubated with compound for approximately 2 min. The voltage pulse protocol was a series of three 150-ms pulses at a frequency of 2.5 Hz. The pre-pulse holding potential was −70 mV, and the steps were to +10, +30 and finally a 200 ms ramp to +50 mV. Voltage was not controlled during compound addition or incubation period.

### Analysis of electrophysiological data

The manual electrophysiological data from oocytes, CHO-K1 cells and DRG neurons were processed and analysed by Clampfit 10.4 (Molecular Devices, LLC.) and GraphPad Prism 5 (GraphPad Software, inc).

#### Analysis of oocyte and CHO-K1 cell recordings

The conductance, *G*_K_(*V*), was calculated as


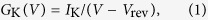


where *I*_K_ is the average steady-state current at the end of a 100-ms pulse, *V* is the absolute membrane voltage, and *V*_rev_ is the reversal potential for K^+^ (set to −80 mV in the oocytes, and calculated to be −89 mV in the CHO-K1 cells). These data were fitted to a Boltzmann equation


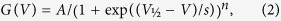


where *A* is amplitude of the curve, *V*_½_ is the midpoint, *s* is the slope, and *n* is an exponent set to 4^19^. The compound-induced *G(V)* shift was determined at the 10% level of the maximum conductance in control solution^19^. The compound-induced *G(V)* shift in CHO-K1 was quantified by subtracting the control *V*_1/2_ from the compound *V*_1/2_ when *n* was set to 1.

For the WT Shaker K channel, if *n* = 1, then *V*_½_ = −24.0 ± 0.9 (*n* = 120) and *s* = 5.6 ± 0.2 (*n* = 31). For the 3R Shaker K channel, *V*_½_ = 25.2 ± 0.3 (*n* = 618) and *s* = 9.5 ± 0.6 (*n* = 40). Despite a difference in slope, there is no correlation between the slope and the shift caused by lipoelectric compounds^17^. This suggests a pure electrostatic effect, where the shift does not depend on the slope of the channel in control solution.

Data for concentration and pH dependent *G(V)* shifts, ∆*V*_G(V)_, in oocytes were fit with a dose-response equation


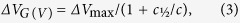


where ∆*V*_max_ is the maximal shift, *c*_½_ is half maximal effective concentration/p*K*_a_ value and *c* is the concentration.

#### Analysis of manual whole-cell recordings of DRG neurons

The compound effect on the resting membrane potential, *V*_m,_ was determined by subtracting the control pre-compound *V*_m_ from the *V*_m_ at the end of the 120 s application of test compound.

#### Analysis of automated planar patch-clamp electrophysiology

Current magnitude was measured automatically from the leak-subtracted traces by the IonWorks^TM^ software by taking the positive peak current recorded during the last 10 ms of the depolarizing steps. Modulation of the Kv-mediated current was assessed by dividing the post-scan Kv current by the respective pre-scan Kv current for each well.

### Statistical analysis

Average values are expressed as mean ± SEM. When comparing compound-induced shifts of mutants with control WT one-way ANOVA together with Dunnett’s multiple comparison test was used. When comparing groups, one-way ANOVA together with Bonferroni’s multiple comparison tests was used. Correlation analysis was done by Pearson’s correlation test and linear regression. P < 0.05 is considered significant for all tests.

### Compound synthesis

The complete description of compound synthesis is found in Supplementary Methods.

## Additional Information

**How to cite this article**: Ottosson, N. E. *et al.* Resin-acid derivatives as potent electrostatic openers of voltage-gated K channels and suppressors of neuronal excitability. *Sci. Rep.* **5**, 13278; doi: 10.1038/srep13278 (2015).

## Supplementary Material

Supplementary Information
