# Supplementary material for: Resin-acid derivatives as potent electrostatic openers of voltage-gated K channels and suppressors of neuronal excitability
Source: Sci Rep. 2015 Aug 24;5:13278. doi: 10.1038/srep13278 (PMC4547393; doi:10.1038/srep13278)
Supplement: Supplementary Information [file srep13278-s1.pdf]

Supplementary information

Resin-acid derivatives as potent electrostatic openers of voltage-gated K channels and suppressors of neuronal excitability

Nina E Ottosson[1], Xiongyu Wu[2], Andreas Nolting[1], Urban Karlsson[1], Per-Eric Lund[1], Katinka Ruda[2], Stefan Svensson[2#], Peter Konradsson[2], Fredrik Elinder[1*]

[1]Department of Clinical and Experimental Medicine, Linköping University, Linköping, Sweden
[2]Department of Physics, Chemistry and Biology, Linköping University, Linköping, Sweden

*e-mail: fredrik.elinder@liu.se
#The work was completed after the death of Stefan Svensson

a

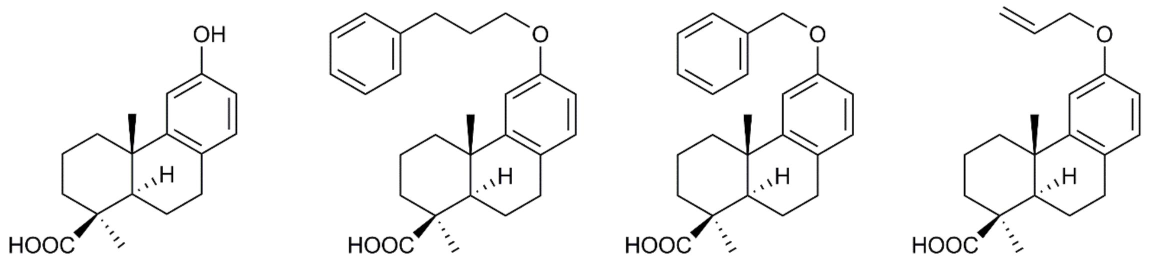

Podocarpic acid (5) **Compound 7** **Compound 8** **Compound 9**

b

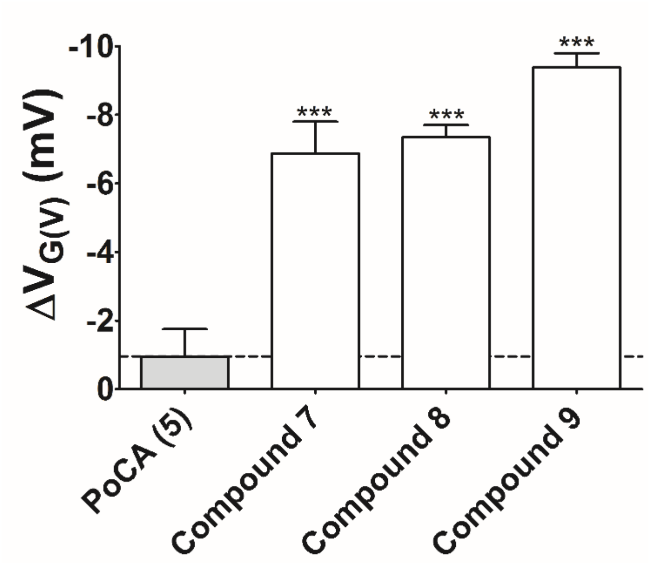

Fig. S1. Efficacy of PoCA-derivates to shift the conductance-versus-voltage, *G*(*V*), curve along the voltage axis (Δ$V_{G(V)}$) for the 3R Shaker channel. (**a**) Molecular structure for PoCA (**5**) and three PoCA-derivates (**Compounds 7, 8, and 9**). (**b**) Compound-induced Δ$V_{G(V)}$ for the 3R Shaker channel. Mean ± SEM (n = 7, 4, 7, and 5; from left to right). The shifts are compared with PoCA (**5**), (one-way ANOVA together with Dunnett's multiple comparison test: ***, $P < 0.001$). **Compound 7** was used for further modifications on the B-ring where allyloxime (**Compound 10**), benzyloxime (**Compound 11**), and propylbenzene-oxime (**Compound 12**) groups were attached to C7. These modifications reduced the potency back to levels close to PoCA (**5**) (**Supplementary Table S1**). Despite these attempts to modify PoCA (**5**) we did not reach the potency of DHAA (**4**) or the other resin acids. Surprisingly the allyloxime attached to C7 in the **Compound 7** background (**Compound 10**) reduced the potency significant (unpaired t-test, $p < 0.05$) while an allyloxime attached to C7 in DHAA (**4**) backgrounds (**Compound 19**, see main article) increased the potency significantly (unpaired t-test, $p < 0.05$). Thus, one but not two hydrophobic groups attached to the B and C rings are beneficial for the effects, suggesting that specific geometries on the hydrophobic end of the molecule are required for a potent lipoelectric effect.

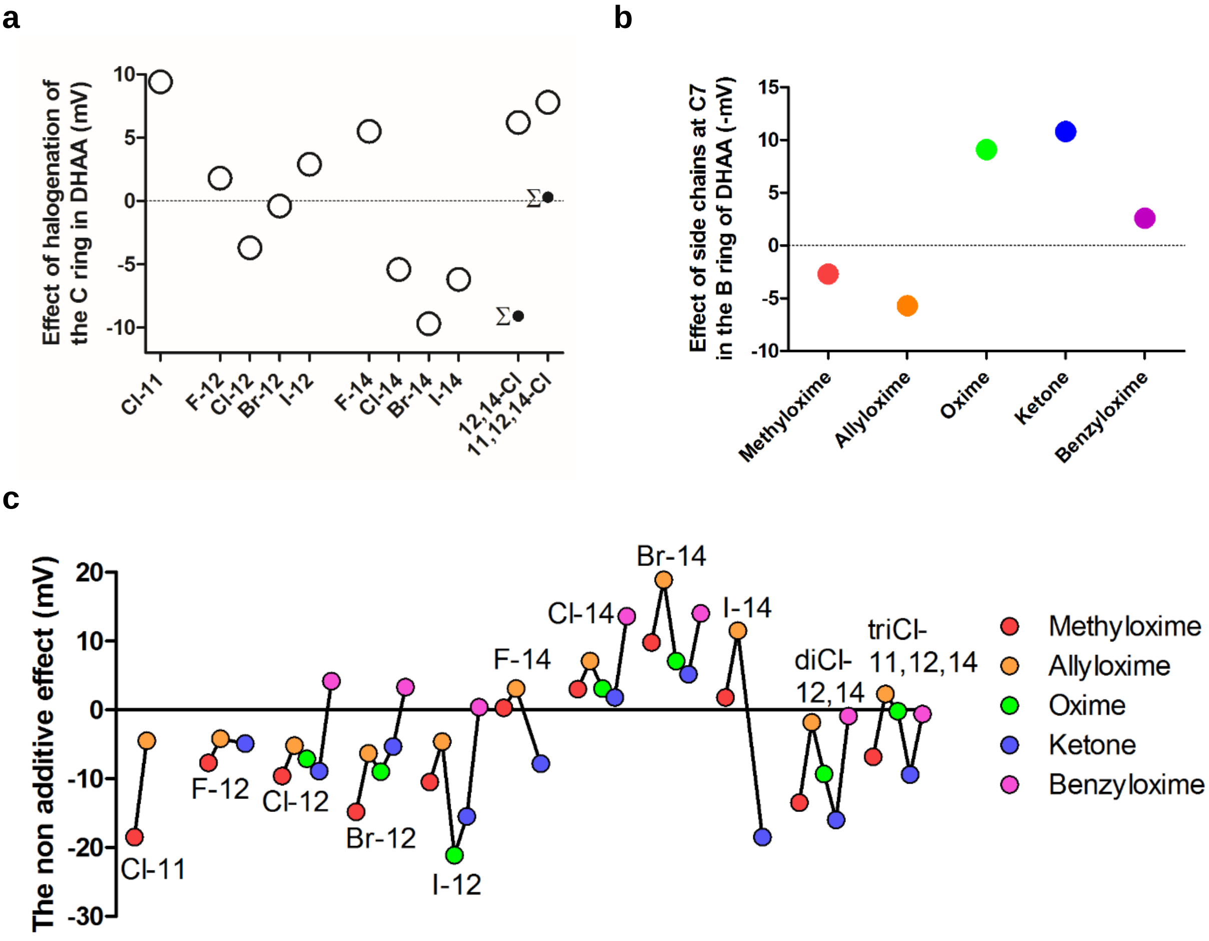

Fig. S2. $\Delta V_{G(V)}$ for derivatives compared to DHAA (4). (**a**) Effects of halogenation introduced in the C ring of DHAA (**4**). The black symbols indicate the expected effect if the effects of the individual chlorinations are additive. (**b**) Effects of side chains introduced to C7 in DHAA (**4**). (**c**) Interaction effects, which is the non-additive component introduced by combining halogenation in the C-ring and a side chain at C7.

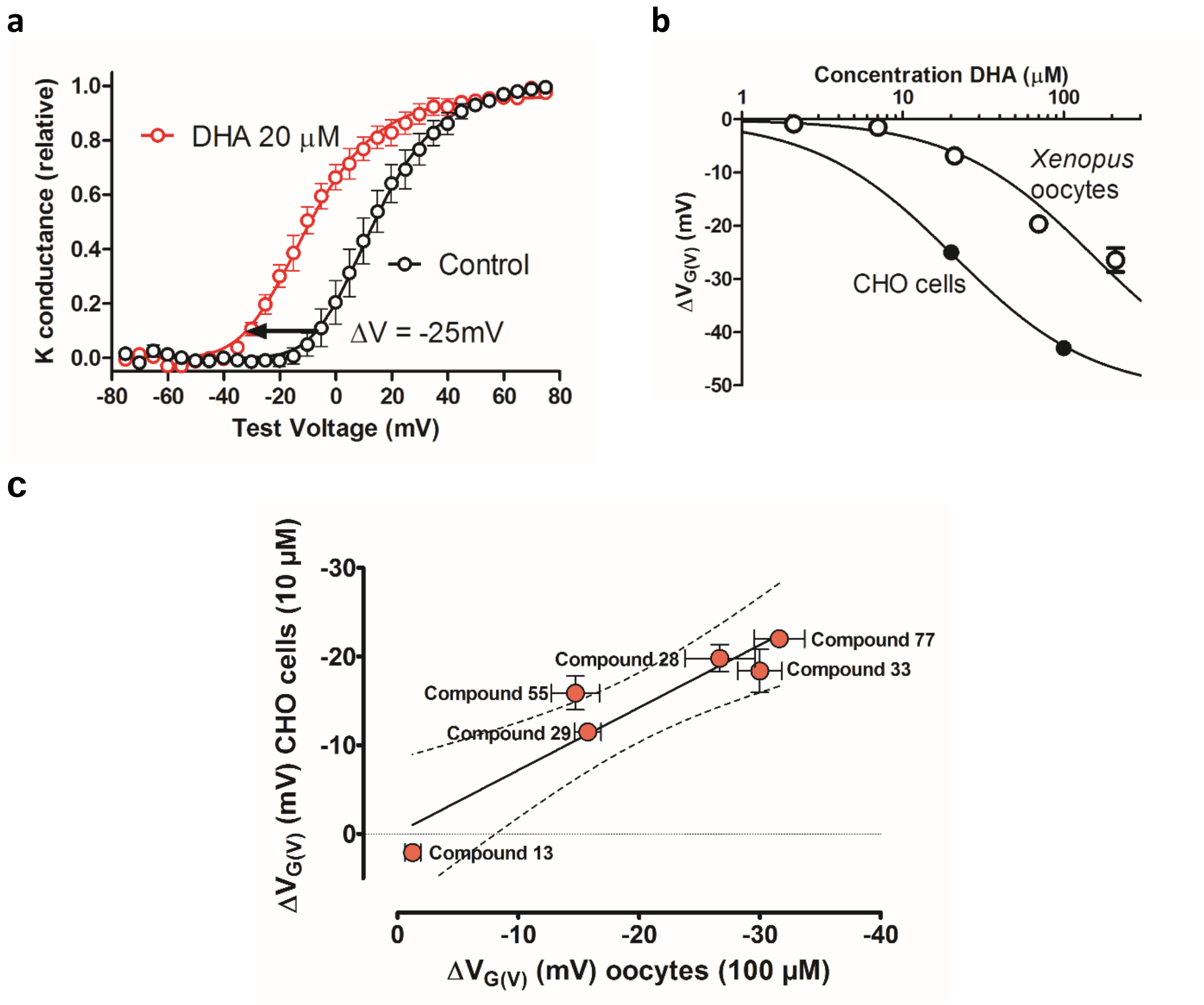

Fig. S3. 3R channel expressed in *Xenopus* oocytes and CHO-cells. (**a**) Effect of 20 μM DHA (**6**) on the 3R channel expressed in CHO cells. (**b**) Dose response curves for DHA-induced $G(V)$ shifts in *Xenopus* oocytes and CHO cells. Data for *Xenopus* oocytes from Ottosson et al. (2014). Data fitted to $\Delta V = \Delta V_{max} / (1 + c_{½} / c)$, where ΔV_{max} was fixed to be equal for both curves. $\Delta V_{max} = -51.4$ mV, $c_{½}$ (*Xenopus*) = 154 μM, and $c_{½}$ (CHO) = 21 μM. (**c**) Correlations between the $G(V)$ shifts for the 3R channel expressed in CHO cells (10 μM at pH 7.4) versus *Xenopus* oocytes (100 μM at pH 7.4). Slope is significantly different from zero (Pearson correlation test and linear regression: $P < 0.01$ for both).

a

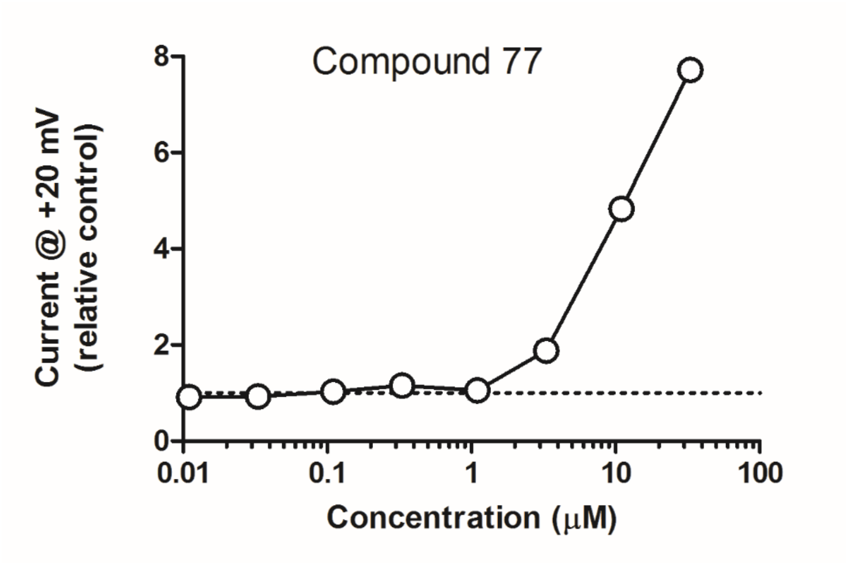

b

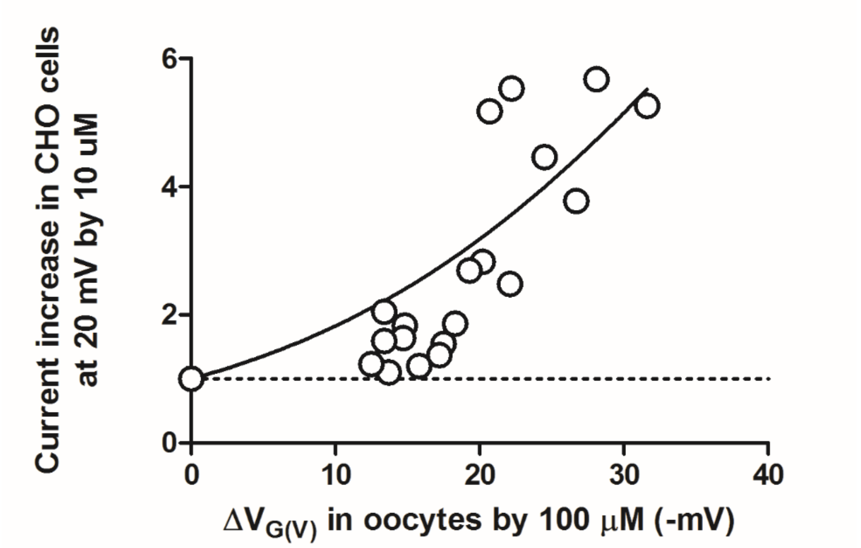

Fig. S4. Effects of resin-acid derivatives on CHO cells measured by the high-throughput Ion Works. (**a**) Concentration-response curve for **Compound 77** measured as relative current increase a +20 mV. (**b**) Relative current increase measured by Ion Works at +20 mV and at 10 μM and pH 7.2. The 20 most potent compounds from the oocyte recordings are compared with the Ion Works data. The following equation, based on a simple Boltzmann equation shifted along the voltage axis, is expected to fit to the data:

$$I_{substance}/I_{control} = (1 + \exp(-(20 - V_{½})/s)) / (1+\exp(-(20 - (V_{½} - \Delta G(V)*0.5))/s)).$$

Parameters for the continuous curve is $V_{½}$ = 40 mV and s = 7.5 mV. The factor 0.5 is to transform oocyte data at 100 μm to CHO cells at 10 μM.

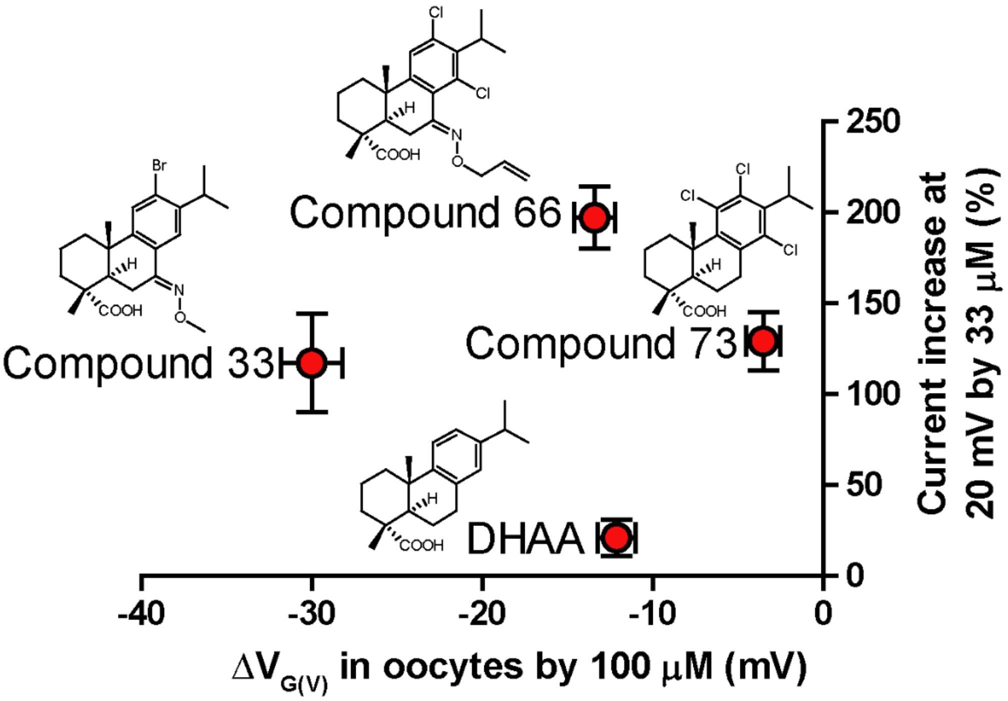

Fig. S5. Comparison of the efficacy of the compounds to increase the current in BK-cells versus to induce a shift of the voltage dependence of activation for the 3R Shaker channel. Data for BK channels from Cui et al. (2010). Error bars indicate SEM (*n* = 4-8).

Name	N:o	Template	C7	C11	C12	C13	C14	n	Shift	SEM
PiMA	1	PiMA	-	-	-	methyl, methylvinyl	-	6	-10.4	0.8
Iso-PiMA	2	Iso-PiMA	-	-	-	methyl, methylvinyl	-	4	-15.9	1.8
AA	3	AA	-	-	-	Isopropyl	-	5	-11.2	2.0
DHAA	4	DHAA	-	-	-	Isopropyl	-	6	-12.1	1.2
PoCA	5	PoCA	-	-	-	-	-	7	-1.0	0.8
DHA	6	DHA	N/A	N/A	N/A	N/A	N/A	15	-19.6	1.0
Cmpd 7	7	PoCA	-	-	A	-	-	4	-6.9	0.9
Cmpd 8	8	PoCA	-	-	B	-	-	7	-7.3	0.4
Cmpd 9	9	PoCA	-	-	C	-	-	5	-9.4	0.4
Cmpd 10	10	PoCA	$=N-O-CH_2-CH=CH_2$	-	A	-	-	5	-4.1	0.7
Cmpd 11	11	PoCA	$=N-O-CH_2-C_6H_5$	-	A	-	-	6	-2.6	0.3
Cmpd 12	12	PoCA	$=N-O-CH_2-CH_2-CH_2-C_6H_5$	-	A	-	-	5	-1.9	0.2
Cmpd 13	13	DHAA	=O	-	-	Isopropyl	-	4	-1.3	0.6
Cmpd 14	14	DHAA	=N-O-H	-	-	Isopropyl	-	4	-3.0	1.3
Cmpd 15	15	DHAA	-OH	-	-	Isopropyl	-	4	-7.3	0.3
Cmpd 16	16	DHAA	$=N-O-CH_2-CH_2-CH_2-C_6H_5$	-	-	Isopropyl	-	5	-3.0	1.0
Cmpd 17	17	DHAA	$=N-O-CH_2-C_6H_5$	-	-	Isopropyl	-	4	-9.5	1.3
Cmpd 18	18	DHAA	$=N-O-CH_3$	-	-	Isopropyl	-	4	-14.8	1.2
Cmpd 19	19	DHAA	$=N-O-CH_2-CH=CH_2$	-	-	Isopropyl	-	4	-17.8	1.9
Cmpd 20	20	DHAA	$=N-O-CH_3$	Cl	-	Isopropyl	-	6	-23.2	0.6
Cmpd 21	21	DHAA	$=N-O-CH_2-CH=CH_2$	Cl	-	Isopropyl	-	4	-12.9	1.4
Cmpd 22	22	DHAA	-	Cl	-	Isopropyl	-	4	-2.7	0.5
Cmpd 23	23	DHAA	$=N-O-CH_3$	-	F	Isopropyl	-	4	-20.7	2.1
Cmpd 24	24	DHAA	$=N-O-CH_2-CH=CH_2$	-	F	Isopropyl	-	4	-20.2	2.0
Cmpd 25	25	DHAA	-	-	F	Isopropyl	-	4	-10.4	2.2
Cmpd 26	26	DHAA	=O	-	F	Isopropyl	-	4	-4.4	0.3
Cmpd 27	27	DHAA	$=N-O-CH_3$	-	Cl	Isopropyl	-	4	-28.1	1.7
Cmpd 28	28	DHAA	$=N-O-CH_2-CH=CH_2$	-	Cl	Isopropyl	-	9	-26.7	2.9
Cmpd 29	29	DHAA	-	-	Cl	Isopropyl	-	4	-15.8	1.1
Cmpd 30	30	DHAA	=N-O-H	-	Cl	Isopropyl	-	5	-13.8	0.4
Cmpd 31	31	DHAA	=O	-	Cl	Isopropyl	-	5	-13.9	0.9
Cmpd 32	32	DHAA	$=N-O-CH_2-C_6H_5$	-	Cl	Isopropyl	-	4	-9.1	1.6
Cmpd 33	33	DHAA	$=N-O-CH_3$	-	Br	Isopropyl	-	8	-30.0	1.8
Cmpd 34	34	DHAA	$=N-O-CH_2-CH=CH_2$	-	Br	Isopropyl	-	5	-24.5	1.2
Cmpd 35	35	DHAA	-	-	Br	Isopropyl	-	4	-12.5	1.8
Cmpd 36	36	DHAA	=N-O-H	-	Br	Isopropyl	-	4	-12.4	1.4
Cmpd 37	37	DHAA	=O	-	Br	Isopropyl	-	4	-7.0	0.8
Cmpd 38	38	DHAA	$=N-O-CH_2-C_6H_5$	-	Br	Isopropyl	-	4	-6.6	0.9
Cmpd 39	39	DHAA	$=N-O-CH_3$	-	I	Isopropyl	-	4	-22.2	2.2
Cmpd 40	40	DHAA	$=N-O-CH_2-CH=CH_2$	-	I	Isopropyl	-	4	-19.3	1.5
Cmpd 41	41	DHAA	-	-	I	Isopropyl	-	6	-9.0	0.7
Cmpd 42	42	DHAA	=N-O-H	-	I	Isopropyl	-	5	-21.0	1.0
Cmpd 43	43	DHAA	=O	-	I	Isopropyl	-	4	-13.7	2.4
Cmpd 44	44	DHAA	$=N-O-CH_2-C_6H_5$	-	I	Isopropyl	-	5	-6.0	1.4
Cmpd 45	45	DHAA	$=N-O-CH_3$	-	-	Isopropyl	F	5	-9.0	1.5
Cmpd 46	46	DHAA	$=N-O-CH_2-CH=CH_2$	-	-	Isopropyl	F	7	-9.2	1.5
Cmpd 47	47	DHAA	-	-	-	Isopropyl	F	4	-6.6	0.7
Cmpd 48	48	DHAA	=O	-	-	Isopropyl	F	4	-3.6	1.7
Cmpd 49	49	DHAA	$=N-O-CH_3$	-	-	Isopropyl	Cl	5	-17.2	1.6

Cmpd 50	50	DHAA	=N-O-CH_2-CH=CH_2	-	-	Isopropyl	Cl	5	-16.1	0.7
Cmpd 51	51	DHAA	-	-	-	Isopropyl	Cl	4	-17.5	3.3
Cmpd 52	52	DHAA	=N-O-H	-	-	Isopropyl	Cl	5	-5.3	0.5
Cmpd 53	53	DHAA	=O	-	-	Isopropyl	Cl	4	-4.9	0.8
Cmpd 54	54	DHAA	=N-O-CH_2-C_6H_5	-	-	Isopropyl	Cl	4	-1.3	0.3
Cmpd 55	55	DHAA	=N-O-CH_3	-	-	Isopropyl	Br	4	-14.7	2.0
Cmpd 56	56	DHAA	=N-O-CH_2-CH=CH_2	-	-	Isopropyl	Br	5	-8.6	0.5
Cmpd 57	57	DHAA	-	-	-	Isopropyl	Br	4	-21.8	0.8
Cmpd 58	58	DHAA	=N-O-H	-	-	Isopropyl	Br	5	-5.6	0.6
Cmpd 59	59	DHAA	=O	-	-	Isopropyl	Br	4	-5.8	0.6
Cmpd 60	60	DHAA	=N-O-CH_2-C_6H_5	-	-	Isopropyl	Br	4	-5.2	1.3
Cmpd 61	61	DHAA	=N-O-CH_3	-	-	Isopropyl	I	4	-19.2	3.1
Cmpd 62	62	DHAA	=N-O-CH_2-CH=CH_2	-	-	Isopropyl	I	4	-12.5	2.1
Cmpd 63	63	DHAA	-	-	-	Isopropyl	I	5	-18.3	0.4
Cmpd 64	64	DHAA	=O	-	-	Isopropyl	I	5	-26.0	1.0
Cmpd 65	65	DHAA	=N-O-CH_3	-	Cl	Isopropyl	Cl	4	-22.1	2.1
Cmpd 66	66	DHAA	=N-O-CH_2-CH=CH_2	-	Cl	Isopropyl	Cl	4	-13.4	1.2
Cmpd 67	67	DHAA	-	-	Cl	Isopropyl	Cl	5	-5.9	0.4
Cmpd 68	68	DHAA	=N-O-H	-	Cl	Isopropyl	Cl	4	-6.1	0.7
Cmpd 69	69	DHAA	=O	-	Cl	Isopropyl	Cl	4	-11.1	1.6
Cmpd 70	70	DHAA	=N-O-CH_2-C_6H_5	-	Cl	Isopropyl	Cl	4	-4.2	0.5
Cmpd 71	71	DHAA	=N-O-CH_3	Cl	Cl	Isopropyl	Cl	4	-13.4	1.9
Cmpd 72	72	DHAA	=N-O-CH_2-CH=CH_2	Cl	Cl	Isopropyl	Cl	5	-7.3	0.5
Cmpd 73	73	DHAA	-	Cl	Cl	Isopropyl	Cl	5	-3.9	1.0
Cmpd 74	74	DHAA	=N-O-H	Cl	Cl	Isopropyl	Cl	4	4.7	0.9
Cmpd 75	75	DHAA	=O	Cl	Cl	Isopropyl	Cl	4	-2.5	1.3
Cmpd 76	76	DHAA	=N-O-CH_2-C_6H_5	Cl	Cl	Isopropyl	Cl	4	-1.9	0.3
Cmpd 77	77	DHAA	-	-	Cl	Cl	Cl	9	-31.6	2.1

Table S1 | Summary of data for compounds tested on the 3R Shaker channel expressed in oocytes. C7, C11, C12, C13 and C14 have side chains according to **Figure 3** in the main article. Abbreviations for side chain 12 for compounds 7-12: A = -O-CH_2-CH_2-CH_2-C_6H_5; B = -O-CH_2-C_6H_5; C = -O-CH_2-CH=CH_2. Shift is the shift along the voltage axis in mV.

Supplementary methods: Compound Syntheses

General Methods and Materials

All the solvents and reagents were used without further distillation or drying. The solution of Cl_2 in CCl_4 was prepared in our lab with a concentration of 0.3 M. Micrawave heated reactions were run in an Initiator instrument from Biotage. Analytical thin-layer chromatography was performed on the Merk silica gel $60F_{254}$ glass-backed plates. Flash chromatography was performed with silica gel 60 (particles size (0.040-0.063 mm). Preparative HPLC was run on a Gilson Unipoint system with a Gemini C18 column (100 x 21.20 mm, 5 micron) under neutral condition using gradient CH_3CN/water as eluent (water phase: 95 : 5 water/CH_3CN, 10 mM NH_4OAc, organic phase 90 : 10 CH_3CN/water, 10 mM NH_4OAc). NMR spectra were recorded on a Varian Avance 300 MHz and/or 500 MHz with solvent indicated. Chemical shift was reported in ppm on the δ scale and referenced to the solvent peak. CF_3COOH (-76.55 ppm) was used as internal or external standard for ^{19}F NMR. Pimaric acid (**1**) and isopimaric acid (**2**) from Alomone Labs, abietic acid (**3**) from Sigma-Aldrich (technical grade, purified before using), dehydroabietic acid (**4**) from BOC Sciences, and podocarpic acid (**5**) from Sigma-Aldrich, DHA (**6**) from Larodan, and they are all with purities more than 95%. Compounds **7-14**[1-2], **16-19**[1], **67**[3], **73**[2], were synthesized using the method described in the literature with more than 95% purity. Compounds **29**[4], **35**[1], **51**[4], **36**[1], **37**[1], **65-66**[1] **68-75**[1] were known and synthesized with a similar method from literature[1,4], described in the general procedure. All known products are with purities more than 95% and gave satisfactory analytical and spectroscopic data corresponding to the reported literature values. Because the compounds **33-34**[1] were important, their analytical data were therefore reported as representatives for other known compounds, which were synthesized with a slightly different but optimal procedure from the literature. The compound **77** is new and elucidated from 1D (^1H and ^{13}CNMR), 2D NMR (HSQC, HMBC and COSY) and HRMS data.

General Procedures

Oxidation of substituted DHAA

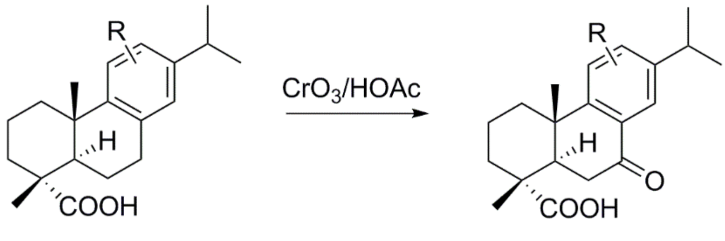

Supplementary Scheme 1. Oxidation of substituted DHAA.

General procedure A: To the solution of helogenated dehydroabietic acid in HOAc added the mixture of CrO_3 (1.2-2.0 equiv) in HOAc at rt, and stirred at 50°C for 3h to overnight, the mixture was concentrated and purified on silica gel with EtOAc/n-heptane/HCOOH (25:75:0.1 to 55:45:0.1) or further with preparative HPLC (20%-90% CH_3CN in water, 10mM NH_4OAc) to give ketone as desired product in 13-59% yield.

Formation of oxime from ketone

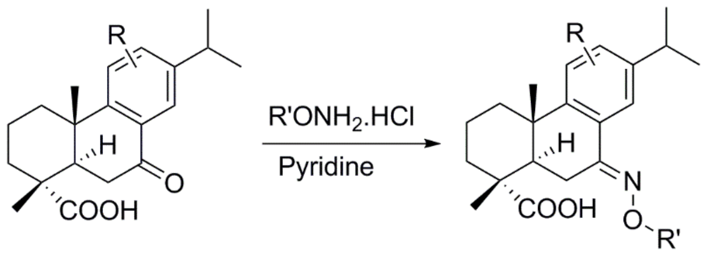

Supplementary Scheme 2. Formation of oxime from ketone.
General procedure B: To the mixture of ketone and 2.0-5.0 equiv R´ONH$_2$ hydrochloride salt added 0.7-1.0 mL EtOH, followed by 2.1-5.1 equiv pyridine, heated under microwave irradiation at 110°C for 1 h, purified on silica gel with 20-45% EtOAc/n-heptane/HCOOH (20:80:0.1 to 45:55:0.1) to give 24-100% yield of oxime as desired product.

Synthesis of compound 15:

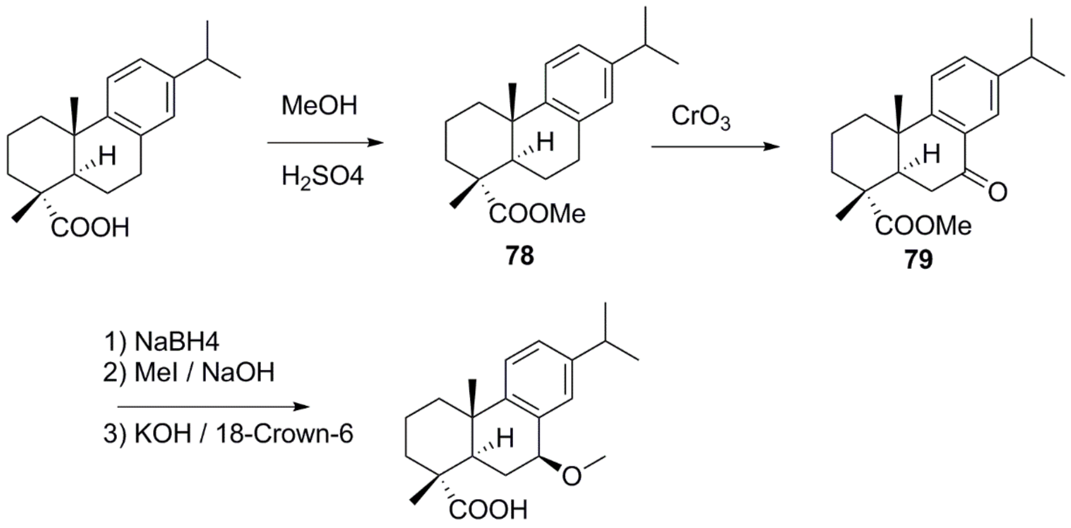

78

79

15

Supplementary Scheme 3. Synthesis of compound **15.**
Methyl (1R,4aS,10aR)-7-isopropyl-1,4a-dimethyl-1,2,3,4,4a,9,10,10a-octahydrophenanthrene-1-carboxylate (**78**): To the solution of dehydroabietic acid (100.0 mg, 0.333 mMol) in 2 mL MeOH added 0.16 mL concentrated H$_2$SO$_4$, sealed tube and heated at 100°C for 36 h, concentrated and purified on silica gel with ethylacetate / n-heptane (10:90 to 20:80) to give ester **78** (80 mg, 76% yield). NMR ^{1}H (500 MHz, CDCl$_3$) δ 7.17 (d, J = 8.5 Hz, 1H), 7.00 (d, J = 8.5 Hz, 1H), 6.88 (br s, 1H), 3.66 (s, 3H), 2.94-2.86 (m, 2H), 2.82 (m, 1H), 2.30 (br d, J = 11.5 Hz, 1H), 2.24 (dd, J = 13.0, 2.0 Hz, 1H), 1.88-1.62 (m, 5H), 1.54-1.46 (m, 1H), 1.44-1.38 (m, 1H), 1.28 (s, 3H), 1.22 (d, J = 7.0 Hz, 6H), 1.21 (s, 3H).
Methyl (1R,4aS,10aR)-7-isopropyl-1,4a-dimethyl-9-oxo-1,2,3,4,4a,9,10,10a-octahydrophenanthrene-1-carboxylate (**79**): Followed the general procedure A, to the resulted methyl ester (80 mg, 0.254 mMol) added CrO$_3$ (30.5 mg, 0.305 mMol) and the mixture was stirred at 50°C for 2 h, then stirred at rt overnight. Purified on silica gel to give product **79** (48.8 mg, 58% yield). NMR ^{1}H (300 MHz, CDCl$_3$) δ 7.87 (d, J = 2.0 Hz, 1H), 7.41 (dd, J = 7.5, 2.0 Hz, 1H), 7.29 (d, J = 7.5 Hz, 1H), 3.65 (s, 3H), 2.93 (m, 1H), 2.75-2.65 (m, 2H),

2.40-2.30 (m, 2H), 1.85-1.77 (m, 3H), 1.76-1.70 (m, 1H), 1.69-1.60 (m, 1H), 1.34 (s, 3H), 1.27-1.22 (m, 9H).

(1R,4aS,9S,10aR)-7-isopropyl-9-methoxy-1,4a-dimethyl-1,2,3,4,4a,9,10,10a-octahydrophenanthrene-1-carboxylic acid (**15**): To the resulted ketone (47.3 mg, 0.151 mMol) from last step in EtOH added $NaBH_4$ (34.2 mg, 0.911 mMol) and stirred at rt for 4 h, concentrated then quenched with water, extracted with EtOAc, dried with $MgSO_4$, filtered and concentrated to give the alcohol. To the resulted alcohol added 1 mL DMF and 18.2 mg 60% NaH (10.8 mg, 0.454 mMol) followed by 28.2 uL MeI (64.2 mg, 0.454 mMol) and stirred overnight. The reaction was quenched with water, extracted with EtOAc, concentrated. To the resulted crude product added KOH (62.0 mg, 1.10 mMol), 18-crown-6 (74.7 mg, 0.283 mMol) followed by 1.5 mL EtOH, irradiated under microwave at 140°C for about 1 h. The reaction mixture was concentrated, then water was added, adjusted PH at about 4, then extracted with EtOAc, concentrated and purified on silica gel with EtOAc/n-heptane (25:75 to 50:50) to give **15** (4.7 mg, yield 9%) and 25.3 mg product mixture of **15** (δ_{H9} 4.58 (dd, J = 9.6, 7.5 Hz, 1H)) and its isomer[5] (δ_{H9} 4.36 (dd, J = 3.6, 2.4 Hz, 1H)) with a ratio of 87:13 according to ^{1}H NMR. **15**: NMR ^{1}H (300 MHz, $CDCl_3$) δ 7.29 (d, J = 1.8 Hz, 1H), 7.15 (d, J = 9.1 Hz, 1H), 7.08 (dd, J = 9.1, 1.8 Hz, 1H), 4.58 (dd, J = 9.6, 7.5 Hz, 1H), 3.44 (s, 3H), 2.87 (m, 1H), 2.39-2.20 (m, 2H), 2.00- 1.65 (m, 7H), 1.32 (s, 3H), 1.30-1.20 (m, 9H). ^{13}C (75 MHz, $CDCl_3$) δ 183.6, 147.2, 146.4, 135.3, 126.1, 125.8, 124.0, 78.5, 55.3, 47.3, 43.0, 38.1, 37.4, 36.8, 33.8, 27.7, 25.4, 24.3, 24.0, 18.5, 16.4. HRMS, calculated mass: 329.2117 [M-H], measured: 329.2130 [M-H],.

Synthesis of compound 20

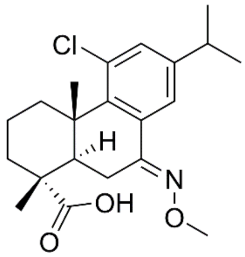

(1R,4aS,10aR)-5-chloro-7-isopropyl-9-(methoxyimino)-1,4a-dimethyl-1,2,3,4,4a,9,10,10a-octahydrophenanthrene-1-carboxylic acid (**20**): Followed the general procedure B, compound **82** (16.9 mg, 0.048 mMol), O-methylhydroxylamine hydrochloride (16.2 mg, 0.194 mMol) and pyridine (16.8 mg, 0.213 mMol) were used and compound **20** (6.1 mg, 33% yield) was achieved. NMR ^{1}H (300 MHz, $CDCl_3$) δ 7.77 (d, J = 1.8 Hz, 1H), 7.18 (d, J = 1.8 Hz, 1H), 3.99 (s, 3H), 3.48 (br d, J = 12.9 Hz, 1H), 2.85 (m, 1H), 2.71 (dd, J = 18.0, 3.9 Hz, 1H), 2.54-2.42 (m, 1H), 2.32 (dd, J = 14.1, 3.3 Hz, 1H), 1.80-1.66 (m, 4H), 1.58-1.48 (m, 1H), 1.35 (s, 3H), 1.32 (s, 3H), 1.24 (d, J = 6.6 Hz, 3H), 1.23 (d, J = 6.3 Hz, 3H). ^{13}C (75 MHz, $CDCl_3$) δ 183.0, 154.6, 147.8, 143.6, 132.7, 131.8, 131.7, 122.1, 62.3, 47.3, 42.6, 40.2, 36.8, 36.3, 33.3, 23.9, 23.5, 18.8, 18.4, 16.7. HRMS calculated mass: 378.1836 [M+H], 380.1806 [M+H+2], measured: 378.1831 [M+H], 378.1810 [M+H+2].

Synthesis of compound 21

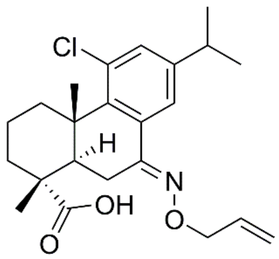

(1R,4aS,10aR)-9-((allyloxy)imino)-5-chloro-7-isopropyl-1,4a-dimethyl-1,2,3,4,4a,9,10,10a-octahydrophenanthrene-1-carboxylic acid (**21**): Followed the general procedure B, compound **82** (8.0 mg, 0.023 mMol), O-allylhydroxylamine hydrochloride (12.6 mg, 0.115 mMol) and pyridine (9.2 mg, 0.117 mMol) were used and compound **21** (2.2 mg, 24% yield) was achieved. NMR ^{1}H (300 MHz, $CDCl_3$) δ 7.76 (d, J = 1.8 Hz, 1H), 7.17 (d, J = 1.8 Hz, 1H), 6.12-5.97 (m, 1H), 5.35-5.26 (m, 1H), 5.26-5.16 (m, 1H), 4.72-4.65 (m, 2H), 3.43 (br d, J = 13.8 Hz, 1H), 2.85 (m, 1H), 2.77 (dd, J = 17.4, 3.6 Hz, 1H), 2.50 (dd, J = 17.4, 13.8 Hz, 1H), 2.34 (dd, J = 13.8, 3.6 Hz, 1H), 1.80-1.67 (m, 4H), 1.58-1.46 (m, 1H), 1.36 (s, 3H), 1.33 (s, 3H), 1.23 (d, J = 6.9 Hz, 3H), 1.22 (d, J = 6.9 Hz, 3H). ^{13}C (75 MHz, $CDCl_3$) δ 182.1, 154.8, 147.8, 143.6, 134.7, 132.8, 132.8, 132.7, 122.2, 117.5, 75.5, 47.2, 42.7, 40.2, 36.8, 33.3, 24.0, 23.9, 23.5, 18.8, 18.4, 16.7. HRMS calculated mass: 404.1993 [M+H], 406.1967 [M+H+2], measured: 404.1987 [M+H], 406.1968 [M+H+2].

Synthesis of compound 22

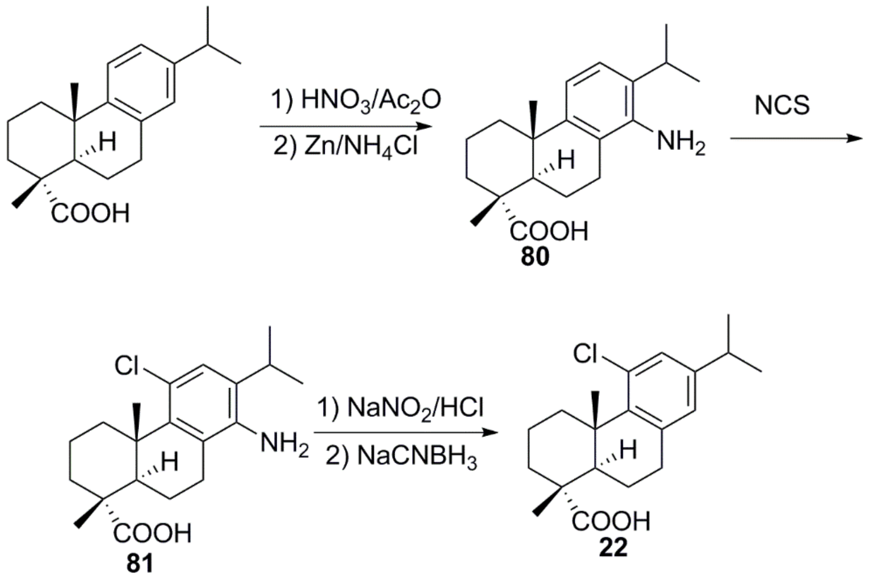

Supplementary Scheme 11. Synthesis of compound **22**.

Step 1. Amination

The reaction mixture of DHAA (400.0 mg, 1.331 mMol) and 183 uL 65% HNO_3 (167.8, 1.663 mMol) in 8.0 mL Ac_2O was stirred at rt overnight, it's fully conversed according to crude HNMR, concentrated to give a raw product for next step without further purification. To the resulted crude product added Zn powder (402.0 mg, 6.149 mMol), 20 mL THF/MeOH

(1:1) and 5.0 mL saturated NH_4Cl aqueous solution, the mixture was stirred at rt for 6 h, full conversion was almost achieved, another 1.0 mL saturated NH_4Cl aqueous solution was added and stirred at rt overnight. Concentrated and 15 mL DCM followed by 15 mL saturated NH_4Cl aqueous solution was added. Dissolved the excess Zn powder with 2N HCl carefully, and then adjusted PH to 6-7 with 1M NaOH aqueous solution. The organic layer was separated, and then the water layer was extracted with DCM 15 mL x 2. The organic layers were combined and concentrated, purified on silica gel with 30-70% EtOAc/n-heptane/HCOOH (30:70:0.1 to 70:30:0.1) and preparative HPLC (20-90% acetonitrile in water, 10mM NH_4OAc) to give compound **80** (106.9 mg, 25% yield). NMR ^1H (300 MHz, $CDCl_3$) δ 7.01 (d, J = 8.1 Hz, 1H), 6.77 (d, J = 8.1 Hz, 1H), 5.09 (br s, 2H), 2.88 (m, 1H), 2.62-2.52 (m, 2H), 2.32-2.20 (m, 2H), 1.90-1.60 (m, 6H), 1.60-1.40 (m, 1H), 1.30-1.15 (m, 12H).

Step2 Chlorination
The reaction mixture of **80** (153.0 mg, 0.485 mMol), N-chlorosuccinimide (66.1 mg, 0.495 mMol) in 2 mL DMF was heated under microwave irradiation at 75°C for 30 min, then purified directly on silica gel without concentration or extraction with EtOAc/n-heptane (30:70 to 50:50) three times to give product **81** (44.0 mg, 26% yield), containing small amount of impurities, which was difficult to be removed. The product was then used without further purification in next step.

Step 3. De-amination
(1R,4aS,10aR)-5-chloro-7-isopropyl-1,4a-dimethyl-1,2,3,4,4a,9,10,10a-octahydrophenanthrene-1-carboxylic acid (**22**): To the compound **81** (39.8 mg, 0.114 mMol) from last step in 5 mL THF added 342 uL 1N HCl followed by $NaNO_2$ (15.7 mg, 0.227 mMol) at 0°C, the mixture was stirred at 0°C until started material disappeared. The freshly prepared solution of $NaCNBH_3$ (71.6 mg, 1.14 mMol) in 1.14 mL water was added to the mixture at 0°C. The temperature was allowed to warm up to room temperature and stirred overnight. About 10 mL DCM was added and the organic layer was separated and concentrated, purified on silica gel with EtOAc/n-heptane/HCOOH (30:70:0.1) to give compound **22** (15.8 mg, 41% yield). The structure was also confirmed with 1D and 2D-NMR. Compound **22**: NMR ^1H (300 MHz, $CDCl_3$) δ 7.01 (d, J = 2.1 Hz, 1H), 6.81 (d, J = 2.1 Hz, 1H), 3.52-3.42 (m, 1H), 3.09-2.91 (m, 1H), 2.85-2.70 (m, 2H), 2.21 (d, J = 10.5 Hz, 1H), 1.82-1.60 (m, 5H), 1.50-1.39 (m, 4H), 1.39-1.23 (m, 4H), 1.23-1.17 (m, 6H). ^{13}C (75 MHz, $CDCl_3$) δ 184.9, 147.2, 142.1, 139.1, 133.1, 128.2, 127.0, 48.4, 47.1, 39.8, 36.4, 35.3, 33.1, 33.0, 23.8, 22.1, 19.9, 18.7, 17.0. HRMS calculated mass: 333.1621 [M-H], 335.1592 [M-H+2], measured: 333.1623 [M-H], 335.1600 [M-H+2].

Synthesis of compound 23

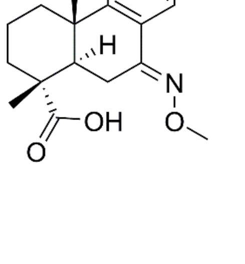

(1R,4aS,10aR)-6-fluoro-7-isopropyl-9-(methoxyimino)-1,4a-dimethyl-1,2,3,4,4a,9,10,10a-octahydrophenanthrene-1-carboxylic acid (**23**): Followed the general procedure B, compound **26** (11.4 mg, 0.034 mMol), O-methylhydroxylamine hydrochloride (5.7 mg, 0.069 mMol) and pyridine (5.7 mg, 0.070 mMol) were used and compound **23** (12.1 mg, 98% yield) was achieved. NMR ^{1}H (300 MHz, $CDCl_3$) δ 7.79 (d, J = 8.1 Hz), 6.87 (d, J = 12.3 Hz, 1H), 4.00 (s, 3H), 3.17 (m, 1H), 2.75-2.52 (m, 2H), 2.20-2.14 (m, 2H), 1.82-1.71 (m, 4H), 1.65-1.54 (m, 1H), 1.36 (s, 3H), 1.27 (d, J = 6.6 Hz, 3H), 1.26 (d, J = 6.9 Hz, 3H), 1.10 (s, 3H). ^{13}C (75 MHz, $CDCl_3$) δ 182.6, 162.0 (d, J_{CF} = 248.7 Hz), 153.4, 150.7 (d, J_{CF} = 6.9 Hz), 133.3 (d, J_{CF} = 16.1 Hz), 125.2 (d, J_{CF} = 3.4 Hz), 124.0 (d, J_{CF} = 5.8 Hz), 110.0 (d, J_{CF} = 24.0 Hz), 62.2, 46.3, 41.6, 37.3, 37.1, 36.6, 27.6, 24.2, 22.8, 22.7, 22.6, 18.1, 16.5. ^{19}F (282.2 MHz, $CDCl_3$) δ -117.5 (dd, J = 12.4, 8.4 Hz). HRMS calculated mass: 362.2132 [M+H], measured: 362.2127 [M+H].

Synthesis of compound 24

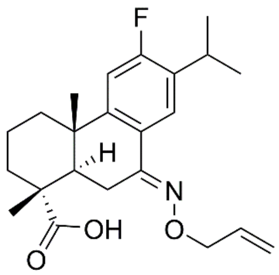

(1R,4aS,10aR)-9-((allyloxy)imino)-6-fluoro-7-isopropyl-1,4a-dimethyl-1,2,3,4,4a,9,10,10a-octahydrophenanthrene-1-carboxylic acid (**24**): Followed the general procedure B, compound **26** (8.7 mg, 0.027 mMol), O-allylhydroxylamine hydrochloride (6.0 mg, 0.055 mMol) and pyridine (4.5 mg, 0.057 mMol) were used and product **24** (10.6 mg, 100% yield) was achieved. NMR ^{1}H (300 MHz, $CDCl_3$) δ 7.78 (d, J = 8.1 Hz), 6.87 (d, J = 11.7 Hz, 1H), 6.17-6.00 (m, 1H), 5.33 (dd, J = 17.7, 1.8 Hz, 1H), 5.23 (dd, J = 11.7, 1.2 Hz, 1H), 4.70 (d, J = 5.4 Hz, 2H), 3.16 (m, 1H), 2.73 (dd, J = 18.9, 5.4 Hz, 1H), 2.60 (dd, J = 18.6, 12.9 Hz, 1H), 2.27 (dd, J = 12.9, 5.4 Hz, 1H), 2.19 (br d, J = 13.5 Hz, 1H), 1.82-1.52 (m, 5H), 1.37 (s, 3H), 1.27 (d, J = 6.6 Hz, 3H), 1.25 (d, J = 6.3 Hz, 3H), 1.11 (s, 3H). ^{13}C (75 MHz, $CDCl_3$) δ 182.2, 162.0 (d, J_{CF} = 246.2 Hz), 153.5, 150.7 (d, J_{CF} = 6.9 Hz), 134.8, 133.4, 125.3 (d, J_{CF} = 3.5 Hz), 124.1 (d, J_{CF} = 5.7 Hz), 117.5, 110.0 (d, J_{CF} = 24.1 Hz), 75.4, 46.3, 41.7, 37.3, 37.2, 36.6, 27.7, 24.3, 22.9, 22.6, 18.1, 16.5. ^{19}F (282.2 MHz, $CDCl_3$) δ -117.5 (dd, J = 11.9, 8.0 Hz). HRMS calculated mass: 388.2288 [M+H], measured: 388.2284 [M+H].

Synthesis of compounds 25 and 47

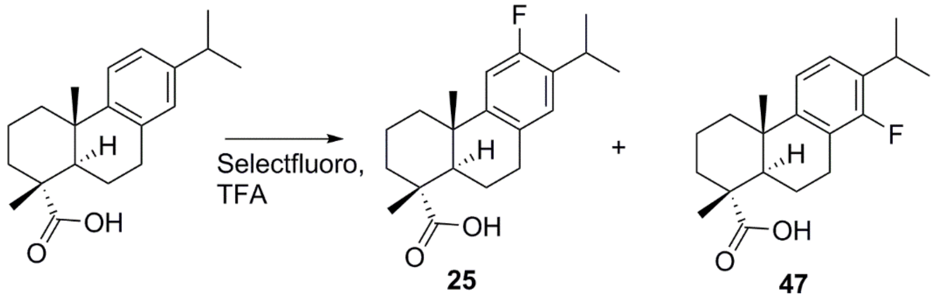

25 **47**

Supplementary Scheme 9. Synthesis of compounds **25** and **47**.
(1R,4aS,10aR)-6-fluoro-7-isopropyl-1,4a-dimethyl-1,2,3,4,4a,9,10,10a-octahydrophenanthrene-1-carboxylic acid (**25**) and (1R,4aS,10aR)-8-fluoro-7-isopropyl-1,4a-dimethyl-1,2,3,4,4a,9,10,10a-octahydrophenanthrene-1-carboxylic acid (**47**): The reaction mixture of DHAA (60.0 mg, 0.166 mMol) and selectfluoro Chloromethyl-4-fluoro-1,4-diazoniabicyclo[2.2.2]octane bis(tetrafluoroborate) (141.5 mg, 0.399 mMol) in 1.2 mL TFA was heated at 100°C for 2 h under microwave irradiation. Three parallel reactions were run instead of running at 180 mg scale because it gave poorer yield at large scale, combined all the reaction mixtures, concentrated and dissolved in DCM, filtered to get rid of the insoluble side product, purified on silica gel with EtOAc/n-heptane/HCOOH (20:80:0.1 to 45:55:0.1), and then further purified with preparative HPLC (35-100% acetonitrile-water-10mM NH_4OAc) to give compound **25** (6.4 mg, yield 3%) and compound **47** (12.5 mg, yield 7%). Compound **25**: NMR ^1H (300 MHz, $CDCl_3$) δ 6.90-6.80 (m, 2H), 3.13 (m, 1H), 2.92-2.79 (m, 2H), 2.26-2.14 (m, 2H), 1.86-1.68 (m, 5H), 1.60-1.51 (m, 2H), 1.28 (s, 3H), 1.26-1.18 (m, 9H). ^{13}C (75 MHz, $CDCl_3$) δ 183.8, 159.3 (d, J_{CF} = 240 Hz), 148.6 (d, J_{CF} = 5.7 Hz), 132.4 (d, J_{CF} = 16.1 Hz), 127.6 (d, J_{CF} = 5.7 Hz), 124.2 (d, J_{CF} = 16.1 Hz), 110.8 (d, J_{CF} = 22.9 Hz), 47.4, 44.6, 38.1, 37.8, 29.4, 27.2, 25.1, 24.1, 22.9, 22.7, 21.9, 18.6, 16.4. ^{19}F (282.2 MHz, $CDCl_3$) δ -124.3 (dd, J = 11.9, 8.4 Hz). HRMS calculated mass: 317.1917 [M-H], measured: 317.1932 [M-H].

Compound **47**: NMR ^1H (300 MHz, $CDCl_3$) δ 7.10-6.92 (m, 2H), 3.19 (m, 1H), 3.00-2.62 (m, 2H), 2.31 (br d, J = 12.9 Hz, 1H), 2.22 (dd, J = 12.3, 1.8 Hz, 1H), 1.90-1.41 (m, 7H), 1.30 (s, 3H), 1.29-1.20 (m, 9H). ^{13}C (75 MHz, $CDCl_3$) δ 185.4, 158.4 (d, J_{CF} = 242.8 Hz), 149.2 (d, J_{CF} = 3.5 Hz), 131.4 (d, J_{CF} = 16.0 Hz), 124.1 (d, J_{CF} = 16.0 Hz), 122.6 (d, J_{CF} = 18.4 Hz), 119.3 (d, J_{CF} = 3.5 Hz), 47.5, 44.2, 38.1, 37.0, 36.8, 27.0 (d, J = 2.3 Hz), 23.1 (d, J = 5.7 Hz), 22.9, 22.7, 20.9, 18.6, 16.3. ^{19}F (282.2 MHz, $CDCl_3$) δ -124.4 (d, J = 7.9 Hz).

Synthesis of compounds 26 and 48

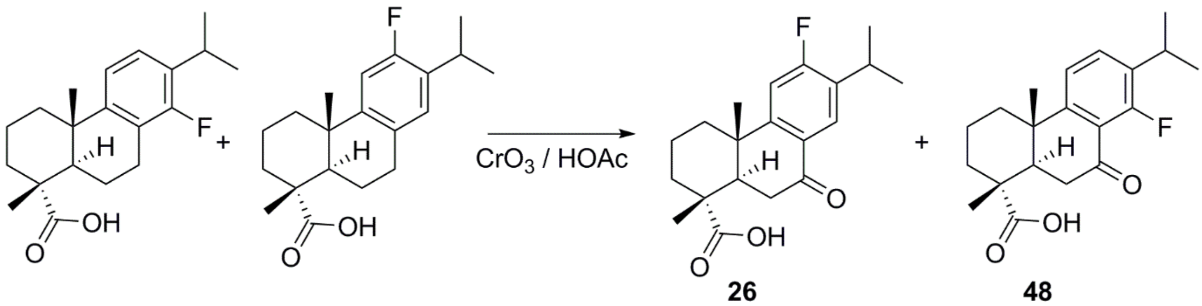

Supplementary Scheme 10. Synthesis of compounds **26** and **48**.
(1R,4aS,10aR)-6-fluoro-7-isopropyl-1,4a-dimethyl-9-oxo-1,2,3,4,4a,9,10,10a-octahydrophenanthrene-1-carboxylic acid (**26**) and (1R,4aS,10aR)-8-fluoro-7-isopropyl-1,4a-dimethyl-9-oxo-1,2,3,4,4a,9,10,10a-octahydrophenanthrene-1-carboxylic acid (**48**): Followed the general procedure A, the mixture of monofluoro-dehydroabietic acid (199.2 mg, 0.626 mMol) and CrO_3 (81.3 mg, 0.814 mMol) were used as started material, the mixture was heated at 50°C overnight, purified with flash chromatography (20-40% EtOAc/n-heptane/HCOOH (20:80:0.1 to 40:60:0.1), then using preparative HPLC to give compound **48** (21.0 mg, yield 10%) and compound **26** (28.3 mg, 14%). Compound **48**: NMR ^1H (300 MHz, $CDCl_3$) δ 7.40 (dd, J = 8.1. 7.5 Hz), 7.09 (d, J = 8.1 Hz, 1H), 3.26 (m, 1H), 2.73-2.50 (m, 3H), 2.31 (br d, J = 11.7 Hz, 1H), 1.86-1.59 (m, 5H), 1.35 (s, 3H), 1.28-1.19 (m, 9H). ^{13}C

(75 MHz, $CDCl_3$) δ 196.7, 183.3, 159.4 (d, J_{CF} = 255.8 Hz), 154.7, 134.6 (d, J_{CF} = 16.1 Hz), 132.5 (d, J_{CF} = 8.0 Hz), 120.1, 118.7 (d, J_{CF} = 4.6 Hz), 46.2, 42.8, 38.8, 37.5, 36.7, 26.8, 23.7, 22.7, 22.5, 18.2, 16.4. ^{19}F (282.2 MHz, $CDCl_3$) δ -119.7 (d, J = 7.9 Hz). HRMS calculated mass: 333.1866 [M+H], measured: 333.1864 [M+H].

Compound **26**: NMR ^{1}H (300 MHz, $CDCl_3$) δ 7.94 (d, J = 8.1 Hz, 1H), 6.96 (d, J = 11.7 Hz, 1H), 3.19 (m, 1H), 2.80-2.60 (m, 2H), 2.48 (dd, J = 12.3, 1.8 Hz, 1H), 2.27 (br d, J = 12.9 Hz, 1H), 1.90-1.58 (m, 5H), 1.35 (s, 3H), 1.29-1.21 (m, 9H). ^{13}C (75 MHz, $CDCl_3$) δ 197.4, 182.8, 164.7 (d, J_{CF} = 253.1 Hz), 155.9 (d, J_{CF} = 8.0 Hz), 134.2 (d, J_{CF} = 16.1), 127.8 (d, J_{CF} = 8.0 Hz), 127.4 (d, J_{CF} = 2.3 Hz), 110.6 (d, J_{CF} = 24.1 Hz), 46.5, 43.8, 37.7, 37.6, 37.1, 36.6, 27.1, 23.6, 22.6, 22.5, 18.2, 16.3. ^{19}F (282.2 MHz, $CDCl_3$) δ -109.5 (dd, J = 11.9, 7.9 Hz). HRMS calculated mass: 333.1866 [M+H], measured: 333.1860 [M+H].

Synthesis of compound 27

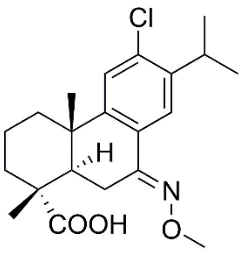

(1R,4aS,10aR)-6-chloro-7-isopropyl-9-methoxyimino-1,4a-dimethyl-1,2,3,4,4a,9,10,10a-octahydrophenanthrene-1-carboxylic acid (**27**): Followed the general procedure B, ketone **31** (21.3 mg, 0.061 mMol), hydroxylamine hydrochloride (10.2 mg, 0.122 mMol) and pyridine (10.1 mg, 0.128 mMol) were used and product **27** (22.3 mg, 97% yield) was achieved. NMR ^{1}H (300 MHz, $CDCl_3$) δ 7.83 (s, 1H), 7.21 (s, 1H), 4.01 (s, 3H), 3.34 (m, 1H), 2.69 (dd, J = 18.6, 5.4 Hz, 1H), 2.58 (dd, J = 18.6, 12.9 Hz, 1H), 2.30-2.19 (m, 2H), 1.80-1.53 (m, 5H), 1.36 (s, 3H), 1.31-1.20 (m, 6H), 1.11 (s, 3H). ^{13}C (75 MHz, $CDCl_3$) δ 183.5, 153.3, 149.7, 143.5, 135.0, 128.1, 124.2, 122.9, 62.3, 46.3, 41.5, 37.3, 37.1, 36.6, 30.2, 24.2, 22.9, 22.8, 22.7, 18.1, 16.5. HRMS calculated mass: 378.1836 [M+H], 380.1806 [M+H+2], measured: 378.1831 [M+H], 378.1810 [M+H+2].

Synthesis of compound 28

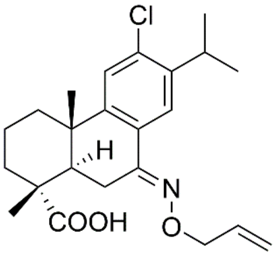

(1R,4aS,10aR)-9-((allyloxy)imino)-6-chloro-7-isopropyl-1,4a-dimethyl-1,2,3,4,4a,9,10,10a-octahydrophenanthrene-1-carboxylic acid (**28**): Followed the general procedure B, ketone **31** (35.4 mg, 0.101 mMol), O-allylhydroxylamine hydrochloride (22.2 mg, 0.203 mMol) and pyridine (16.9 mg, 0.213 mMol) were used and product **28** (30.7 mg, 75% yield) was

achieved. NMR ^{1}H (500 MHz, $CDCl_3$) δ 7.81 (s, 1H), 7.21 (s, 1H), 6.15-6.01 (m, 1H), 5.34 (dd, J = 17.0, 1.5 Hz, 1H), 5.24 (d, J = 10.0 Hz, 1H), 4.74-4.65 (m, 2H), 3.33 (m, 1H), 2.71 (dd, J = 19.0, 5.0 Hz, 1H), 2.61 (dd, J = 19.0, 14.0 Hz, 1H), 2.40-2.19 (m, 2H), 1.80-1.55 (m, 5H), 1.36 (s, 3H), 1.27 (d, J = 7.0 Hz, 3H), 1.24 (d, J = 7.0 Hz, 3H), 1.11 (s, 3H). ^{13}C (125 MHz, $CDCl_3$) δ 184.0, 153.5, 149.7, 143.5, 135.0, 134.7, 128.2, 122.9, 117.6, 75.5, 46.3, 41.5, 37.3, 37.1, 36.6, 30.2, 24.3, 22.9, 22.8, 22.7, 18.1, 16.5. HRMS calculated mass: 404.1993 [M+H], 406.1967 [M+H+2], measured: 404.1987 [M+H], 406.1968 [M+H+2].

Synthesis of compounds 29 and 51:

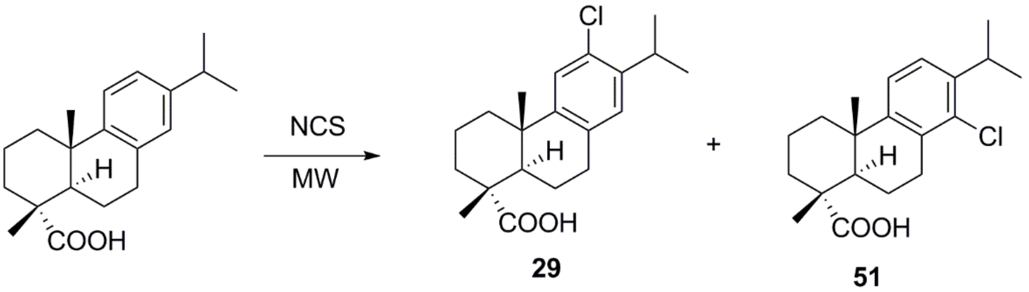

Supplemetary Scheme 4. Synthesis of compounds **29** and **51**.

(1R,4aS,10aR)-6-chloro-7-isopropyl-1,4a-dimethyl-1,2,3,4,4a,9,10,10a-octahydrophenanthrene-1-carboxylic acid (**29**) and (1R,4aS,10aR)-8-chloro-7-isopropyl-1,4a-dimethyl-1,2,3,4,4a,9,10,10a-octahydrophenanthrene-1-carboxylic acid (**51**): The mixture of dehydroabietic acid (300.4 mg, 1.0 mMol) and N-chlorosuccinimide (106.8 mg, 0.8 mMol) in 3 mL acetonitrile in a 2-5.0 mL vial was heated under microwave irradiation at 100°C for 20 min, then another 106.8 mg of NCS was added and stirred at 100°C for another 30 min. Concentrated and purified on silica gel with EtOAc/n-heptane/HCOOH (20:80:0.1 to 40:60:0.1) to give **51** (51.6 mg, 15% yield) and **29** (139.0 mg, yield 42%) as white solid.
Compound **29**: NMR ^{1}H (300 MHz, $CDCl_3$) δ 7.19 (s, 1H), 6.94 (s, 1H), 3.32, (m, 1H), 2.95-2.80 (m, 2H), 2.26 (d, J = 12.9 Hz, 1H), 2.20 (dd, J = 12.3, 2.4 Hz, 1H), 1.90-1.63 (m, 5H), 1.62-1.42 (m, 2H), 1.29 (s, 3H), 1.26-1.19 (m, 9H). ^{13}C (75 MHz, CDCl3) δ 185.5, 148.4, 142.6, 133.8, 130.8, 127.2, 125.3, 47.5, 44.4, 37.9, 37.1, 36.8, 29.8, 29.6, 25.1, 22.9, 22.8, 21.7, 18.5, 16.3.
Compound **51**: NMR ^{1}H (300 MHz, $CDCl_3$) δ 7.17 (d, J = 8.1 Hz, 1H), 7.11 (d, J = 8.1 Hz, 1H), 3.49-3.36 (m, 1H), 3.04-2.92 (m, 1H), 2.90-2.71 (m, 1H), 2.30 (br d, J = 12.3 Hz, 1H), 2.19 (dd, J = 10.5, 2.4 Hz, 1H), 1.92-1.59 (m, 6H), 1.52-1.40 (m, 1H), 1.29 (s, 3H), 1.27-1.16 (m, 9H). ^{13}C (75 MHz, $CDCl_3$) δ 184.8, 148.9, 143.0, 133.8, 133.4, 123.7, 122.7, 47.4, 44.0, 38.3, 37.3, 36.7, 30.3, 29.3, 25.2, 23.0, 22.7, 21.6, 18.7, 16.3. ,

Synthesis of compound 30

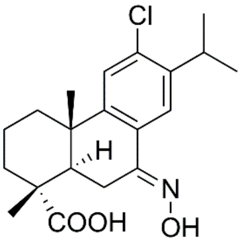

(1R,4aS,10aR)-6-chloro-7-isopropyl-9-hydroxyimino-1,4a-dimethyl-1,2,3,4,4a,9,10,10a-octahydrophenanthrene-1-carboxylic acid (**30**): Followed the general procedure B, ketone **31** (14.3 mg, 0.041 mMol), hydroxylamine hydrochloride (5.7 mg, 0.082 mMol) and pyridine (6.8 mg, 0.086 mMol) were used and product **30** (9.0 mg, 60% yield) was achieved. NMR ^{1}H (300 MHz, $CDCl_3$) δ 7.68 (s, 1H), 7.24 (s, 1H), 3.30 (m, 1H), 2.92-2.63 (m, 2H), 2.32 (dd, J = 12.9, 4.8 Hz, 1H), 2.25 (br d, J = 12.3 Hz, 1H), 1.84-1.72 (m, 4H), 1.67-1.56 (m, 1H), 1.39 (s, 3H), 1.18 (d, J = 6.9 Hz, 3H), 1.16 (d, J = 6.6 Hz, 3H), 1.12 (s, 3H). ^{13}C (75 MHz, $CDCl_3$) δ 183.2, 155.8, 150.6, 144.1, 136.4, 126.4, 124.6, 123.4, 46.1, 41.4, 37.1, 36.8, 30.1, 24.5, 22.9, 22.7, 22.6, 18.1, 16.6. HRMS calculated mass: 364.1680 [M+H], 366.1650 [M+H+2], measured: 364.1673 [M+H], 366.1651 [M+H+2].

Synthesis of compound 31

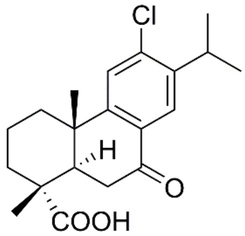

(1R,4aS,10aR)-6-chloro-7-isopropyl-9-oxo-1,4a-dimethyl-1,2,3,4,4a,9,10,10a-octahydrophenanthrene-1-carboxylic acid (**31**). Followed the general procedure A, compound **29** (83.0 mg, 0.248 mMol) and CrO_3 (29.7 mg, 0.297 mMol) were used as started materials, and compound **31** (37.0 mg, yield 43%) was got. NMR ^{1}H (300 MHz, $CDCl_3$) δ 7.94 (s, 1H), 7.33 (s, 1H), 3.54 (m, 1H), 2.78-2.61 (m, 2H), 2.49 (d, J = 9.0 Hz, 1H), 2.31 (d, J = 7.5 Hz, 1H), 1.86-1.73 (m, 4H), 1.70-1.59 (m, 1H), 1.35 (s, 3H), 1.30-1.20 (m, 9H). ^{13}C (75 MHz, $CDCl_3$) δ 197.9, 183.3, 154.0, 144.3, 140.1, 129.6, 126.1, 125.0, 46.5, 43.6, 37.8, 37.5, 37.1, 36.6, 30.1, 23.7, 22.7, 22.6, 18.1, 16.3. HRMS calculated mass: 349.1571 [M+H], 351.1541[M+H+2], measured: 349.1567 [M+H], 351.1545 [M+H+2].

Synthesis of compound 32

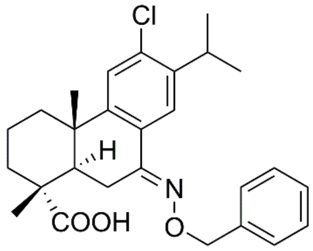

(1R,4aS,10aR)-9-((benzyloxy)imino)-6-chloro-7-isopropyl-1,4a-dimethyl-1,2,3,4,4a,9,10,10a-octahydrophenanthrene-1-carboxylic acid (**32**): Followed the general procedure B, ketone **31** (14.0 mg, 0.040 mMol), O-benzylhydroxylamine hydrochloride (12.8 mg, 0.080 mMol) and pyridine (6.7mg, 0.084 mMol) were used and compound **32** (10.6 mg, 58% yield) was achieved. NMR ^{1}H (500 MHz, $CDCl_3$) δ 7.82 (s, 1H), 7.44-7.27 (m, 5H),

7.20 (s, 1H), 5.23 (s, 2H), 3.33 (m, 1H), 2.76 (dd, J = 18.6, 5.4 Hz, 1H), 2.60 (dd, J = 18.9, 13.5 Hz, 1H), 2.30-2.18 (m, 2H), 1.79-1.54 (m, 5H), 1.35 (s, 3H), 1.27 (d, J = 6.3 Hz, 3H), 1.23 (d, J = 6.9 Hz, 3H), 1.10 (s, 3H). ^{13}C (75 MHz, $CDCl_3$) δ 183.7, 153.7, 149.7, 143.4, 138.2, 135.0, 128.5, 128.4, 128.2, 127.9, 124.2, 123.0, 76.5, 46.3, 41.5, 37.3, 37.1, 36.6, 30.1, 24.4, 22.9, 22.8, 22.7, 18.1, 16.5. HRMS calculated mass: 454.2149 [M+H], 456.2120 [M+H+2], measured: 454.2143 [M+H], 456.2128 [M+H+2].

Synthesis of compound 33

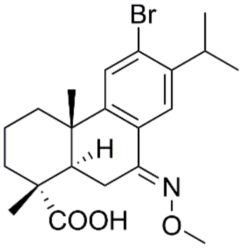

(1R,4aS,10aR)-6-bromo-7-isopropyl-9-(methoxyimino)-1,4a-dimethyl-1,2,3,4,4a,9,10,10a-octahydrophenanthrene-1-carboxylic acid (**33**): Followed the general procedure B, ketone **37** (22.0 mg, 0.056 mMol), O-methylhydroxylamine hydrochloride (9.4 mg, 0.112 mMol) and pyridine (9.3 mg, 0.118 mMol) were used and product **33** (17.4 mg, 74% yield) was achieved. NMR ^{1}H (300 MHz, $CDCl_3$) δ 7.80 (s, 1H), 7.40 (s, 1H), 4.01 (s, 3H), 3.30 (m, 1H), 2.75-2.48 (m, 2H), 2.30-2.20 (m, 2H), 1.80-1.55 (m, 5H), 1.35 (s, 3H), 1.27 (d, J = 6.9 Hz, 3H), 1.24 (d, J = 6.6 Hz, 3H), 1.10 (s, 3H). ^{13}C (75 MHz, $CDCl_3$) δ 183.9, 153.4, 149.9, 145.2, 128.8, 127.5, 126.2, 122.8, 62.3, 46.3, 41.4, 37.3, 37.1, 36.6, 32.8, 24.2, 23.0, 22.9, 18.1, 16.5.

Synthesis of compound 34

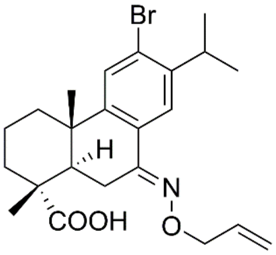

(1R,4aS,10aR)-6-bromo-9-((allyloxy)imino)-7-isopropyl-1,4a-dimethyl-1,2,3,4,4a,9,10,10a-octahydrophenanthrene-1-carboxylic acid (**34**): Followed the general procedure B, ketone **37** (22.0 mg, 0.056 mMol), O-allylhydroxylamine hydrochloride (12.3 mg, 0.112 mMol) and pyridine (9.3 mg, 0.118 mMol) were used and product **34** (15.7 mg, 63% yield) was achieved. NMR ^{1}H (500 MHz, $CDCl_3$) δ 7.79 (s, 1H), 7.40 (s, 1H), 6.13-6.02 (m, 1H), 5.33 (d, J = 17.0 Hz, 1H), 5.23 (d, J = 11.0 Hz, 1H), 4.72 (d, J = 6.0 Hz, 2H), 3.29 (m, 1H), 2.71 (dd, J = 19.0, 5.0 Hz, 1H), 2.61 (dd, J = 18.5, 13.5 Hz, 1H), 2.28-2.19 (m, 2H), 1.80-1.71 (m, 4H), 1.66-1.57 (m, 1H), 1.35 (s, 3H), 1.26 (d, J = 7.0 Hz, 3H), 1.23 (d, J = 7.0 Hz, 3H), 1.11 (s, 3H). ^{13}C (75 MHz, $CDCl_3$) δ 182.1, 153.5, 149.9, 134.7, 128.9, 127.5, 126.1, 122.9, 117.6, 75.6, 46.2, 41.6, 37.3, 37.2, 36.6, 32.8, 24.3, 23.0, 22.9, 18.1, 16.5.

Synthesis of compound 35

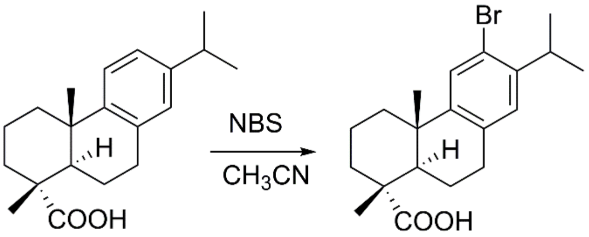

35

Supplementary Scheme 6. Synthesis of compound **35.**

(1R,4aS,10aR)-6-bromo-7-isopropyl-1,4a-dimethyl-1,2,3,4,4a,9,10,10a-octahydrophenanthrene-1-carboxylic acid (**35**): The mixture of dehydroabietic acid (400.0 mg, 1.332 mMol) and N-bromosuccinimide (189.6 mg, 1.065 mMol) in 3 mL acetonitrile in a 2.0-5.0 mL vial was heated under microwave irradiation at 90°C for 30 min, the conversion was not completed according to HPLC, then another 189.6 mg of N-bromosuccinimide was added and stirred at 90°C for another 30 min. Full conversion was not achieved either, 189.6 mg NBS was added again and heated under microwave at 90°C for another 30 min, this time full conversion was achieved. Concentrated and purified on silica gel using EtOAc/n-heptane/HCOOH (20:80:0.1 to 45:55:0.1) to give compound **35** (252.0 mg, 50% yield) as white solid. NMR ^{1}H (300 MHz, $CDCl_3$) δ 7.37 (s, 1H), 6.92, (s, 1H), 3.27 (m, 1H), 2.96-2.81 (m, 2H), 2.26 (br d, J = 12.9 Hz, 1H), 2.19 (dd, J = 12.3, 1.8 Hz, 1H), 1.84-1.68 (m, 6H), 1.60-1.48 (m, 1H), 1.28 (s, 3H), 1.26-1.17 (m, 9H). ^{13}C (75 MHz, $CDCl_3$) δ 185.4, 148.9, 144.2, 134.6, 128.6, 127.3, 121.6, 47.5, 44.4, 37.9, 37.1, 36.8, 32.5, 29.6, 25.1, 23.1, 22.9, 21.7, 18.5, 16.3.

Synthesis of compound 36

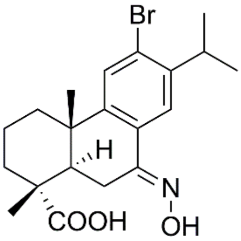

(1R,4aS,10aR)-6-bromo-9-(hydroxyimino)-7-isopropyl-1,4a-dimethyl-1,2,3,4,4a,9,10,10a-octahydrophenanthrene-1-carboxylic acid (**36**): Followed the general procedure B, ketone **37** (14.0 mg, 0.036 mMol), hydroxylamine hydrochloride (4.9 mg, 0.071 mMol) and pyridine (5.9 mg, 0.075 mMol) were used and product **36** (9.1 mg, 63% yield) was achieved. NMR ^{1}H (300 MHz, $CDCl_3$) δ 7.64 (s, 1H), 7.43 (s, 1H), 3.26 (m, 1H), 2.86 (dd, J = 19.2, 5.4 Hz, 1H), 2.70 (dd, J = 19.2, 13.5 Hz, 1H), 2.32 (dd, J = 13.5, 5.1 Hz, 1H), 2.24 (br d, J = 11.7 Hz, 1H), 1.85-1.71 (m, 4H), 1.69-1.55 (m, 1H), 1.38 (s, 3H), 1.17 (d, J = 6.3 Hz, 3H), 1.16 (d, J = 6.6 Hz, 3H), 1.12 (s, 3H). ^{13}C (75 MHz, $CDCl_3$) δ 183.1, 155.8, 150.8, 145.7, 128.0, 127.5, 123.2, 46.1, 41.5, 37.2, 36.8, 32.8, 24.5, 23.0, 22.9, 22.8, 18.1, 16.6.

Synthesis of compound 37

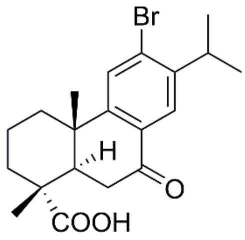

(1R,4aS,10aR)-6-bromo-7-isopropyl-9-oxo-1,4a-dimethyl-1,2,3,4,4a,9,10,10a-octahydrophenanthrene-1-carboxylic acid (**37**): Followed the general procedure A, compound **35** (222.0 mg, 0.585 mMol) and CrO_3 (70.2 mg, 0.702 mMol) were used as started material, the mixture was heated at 50°C for 3h, purified and compound **37** was got (118.0 mg, yield 51%). NMR ^1H (300 MHz, $CDCl_3$) δ 7.90 (s, 1H), 7.53 (s, 1H), 3.32, (m, 1H), 2.80-2.62 (m, 2H), 2.49 (d, J = 13.8 Hz, 1H), 2.31 (br d, J = 12.9 Hz, 1H), 1.85-1.76 (m, 4H), 1.70-1.60 (m, 1H), 1.34 (s, 3H), 1.27 (s, 3H), 1.26 (d, J = 7.5 Hz, 3H), 1.23 (d, J = 6.9 Hz, 3H). ^{13}C (75 MHz, $CDCl_3$) δ 198.0, 182.9, 154.0, 146.0, 131.7, 130.2, 128.4, 125.9, 46.5, 43.6, 37.8, 37.4, 37.1, 36.6, 32.8, 23.7, 22.9, 22.7, 18.1, 16.3.

Synthesis of compound 38

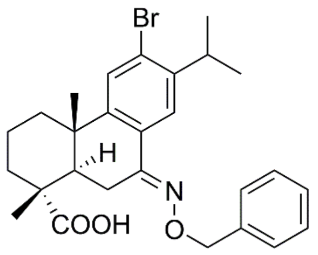

(1R,4aS,10aR)-9-((benzyloxy)imino)-6-bromo-7-isopropyl-1,4a-dimethyl-1,2,3,4,4a,9,10,10a-octahydrophenanthrene-1-carboxylic acid (**38**): Followed the general procedure B, ketone **37** (25.0 mg, 0.064 mMol), O-benzylhydroxylamine hydrochloride (20.3 mg, 0.127 mMol) and pyridine (10.6 mg, 0.134 mMol) were used and product **38** (19.7 mg, 62% yield) was got. NMR ^1H (300 MHz, $CDCl_3$) δ 7.80 (s, 1H), 7.45-7.27 (m, 6H), 5.23 (s, 2H), 3.29 (m, 1H), 2.75 (dd, J = 18.6, 5.1 Hz, 1H), 2.60 (dd, J = 18.6, 12.9 Hz, 1H), 2.29-2.18 (m, 2H), 1.80-1.52 (m, 5H), 1.35 (s, 3H), 1.26 (d, J = 6.9 Hz, 3H), 1.23 (d, J = 6.9 Hz, 3H), 1.10 (s, 3H). ^{13}C (75 MHz, $CDCl_3$) δ 183.1, 153.8, 149.9, 145.1, 138.2, 128.8, 128.5, 128.4, 127.9, 127.5, 126.2, 123.0, 76.6, 46.3, 41.5, 37.3, 37.1, 36.6, 32.8, 24.4, 23.0, 22.9, 18.1, 16.5. HRMS calculated mass: 498.1644 [M+H], 500.1623 [M+H+2], measured: 498.1638 [M+H], 500.1622 [M+H+2].

***Synthesis of compound 3*9**

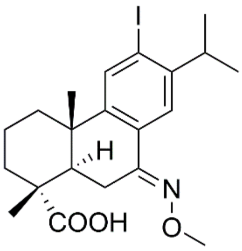

(1R,4aS,10aR)-6-iodo-7-isopropyl-9-(methoxyimino)--1,4a-dimethyl-1,2,3,4,4a,9,10,10a-octahydrophenanthrene-1-carboxylic acid (**39**): Followed the general procedure B, ketone **43** (20.0 mg, 0.045 mMol), O-methylhydroxylamine hydrochloride (7.6 mg, 0.091 mMol) and pyridine (7.5 mg, 0.095 mMol) were used and compound **39** (16.3 mg, 76% yield) was achieved. NMR ^{1}H (300 MHz, $CDCl_3$) δ 7.75 (s, 1H), 7.67 (s, 1H), 4.01 (s, 3H), 3.12 (m, 1H), 2.70-2.50 (m, 2H), 2.28-2.17 (m, 2H), 1.80-1.70 (m, 4H), 1.66-1.55 (m, 1H), 1.35 (s, 3H), 1.26 (d, J = 6.9 Hz, 3H), 1.23 (d, J = 6.9 Hz, 3H), 1.10 (s, 3H). ^{13}C (75 MHz, $CDCl_3$) δ 183.4, 153.5, 150.1, 148.3, 134.4, 129.7, 121.9, 103.4, 62.3, 46.3, 41.4, 38.0, 37.3, 37.1, 36.4, 24.2, 23.3, 23.2, 22.9, 18.1, 16.5. HRMS calculated mass: 470.1192 [M+H], measured: 470.1187 [M+H].

Synthesis of compound 40

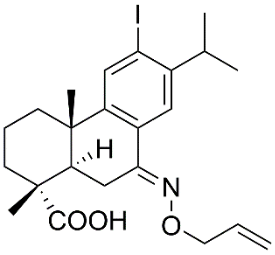

(1R,4aS,10aR)-9-((allyloxy)imino)-6-iodo-7-isopropyl-1,4a-dimethyl-1,2,3,4,4a,9,10,10a-octahydrophenanthrene-1-carboxylic acid (**40**): Followed the general procedure B, ketone **43** (29.5 mg, 0.067 mMol), O-allylhydroxylamine hydrochloride (14.7 mg, 0.134 mMol) and pyridine (11.1 mg, 0.141 mMol) were used and product **40** (28.3 mg, 85% yield) was achieved. NMR ^{1}H (300 MHz, $CDCl_3$) δ 7.74 (s, 1H), 7.67 (s, 1H), 6.18-6.01 (m, 1H), 5.33 (dd, J = 17.1, 1.8 Hz, 1H), 5.23 (dd, J = 10.5, 1.5 Hz, 1H), 4.75-4.69 (m, 2H), 3.12 (m, 1H), 2.78-2.55 (m, 2H), 2.30-2.18 (m, 2H), 1.81-1.53 (m, 5H), 1.35 (s, 3H), 1.25 (d, J = 6.3 Hz, 3H), 1.22 (d, J = 6.6 Hz, 3H), 1.11 (s, 3H). ^{13}C (75 MHz, $CDCl_3$) δ 183.6, 153.7, 150.2, 148.2, 134.7, 134.4, 129.8, 122.0, 117.6, 103.4, 75.6, 46.3, 41.4, 37.9, 37.3, 37.1, 36.4, 24.3, 23.3, 23.1, 23.0, 18.1, 16.5. HRMS calculated mass: 496.1349 [M+H], measured: 496.1343 [M+H].

Synthesis of compound 41 and compound 63

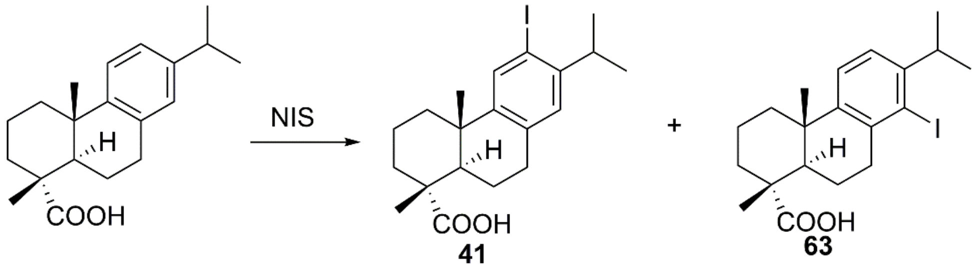

Supplementary Scheme 8. Synthesis of compounds **41** and **63.**

(1R,4aS,10aR)-6-iodo-7-isopropyl-1,4a-dimethyl-1,2,3,4,4a,9,10,10a-octahydrophenanthrene-1-carboxylic acid (**41**) and (1R,4aS,10aR)-8-iodo-7-isopropyl-1,4a-dimethyl-1,2,3,4,4a,9,10,10a-octahydrophenanthrene-1-carboxylic acid (**63**): The mixture of dehydroabietic acid (500.0 mg, 1.664 mMol), N-iodosuccinimide (243.0 mg, 1.080 mMol) and 0.76 mL TFA (1.130 g, 11.648 mMol) in 10 mL acetonitrile in a 10.0-20.0 mL vial was heated under microwave irradiation at 90°C for 60 min, then another part of N-iodosuccinimide (218.0 mg, 0.969 mMol) was added and irradiated again under microwave for another 60 min at 90°C. Full conversion was achieved and the crude HNMR was recorded, the ratio of the 14IDHAA and 12IDHAA was about 0.2 : 1 according to HNMR. Concentrated and purified on silica gel with EtOAc/n-heptane/HCOOH (2080:0.1 to 40:60:0.1) and using preparative HPLC (70%-90% acetonitrile in water, 10 M NH_4OAc) to give compound **63** (56.6 mg, yield 8%) and compound **41** (345.6 mg, yield 55%). Compound **41**: NMR ^{1}H (300 MHz, $CDCl_3$) δ 7.64 (s, 1H), 6.89 (s, 1H), 3.09 (m, 1H), 2.91-2.82 (m, 2H), 2.25 (br d, J = 12.9 Hz, 1H), 2.19 (dd, J = 12.3, 2.4 Hz, 1H), 1.90-1.70 (m, 5H), 1.60-1.45 (m, 2H), 1.28 (s, 3H), 1.24-1.16 (m, 9H). ^{13}C (75 MHz, $CDCl_3$) δ 185.4, 149.2, 147.3, 135.6, 135.5, 126.6, 98.2, 47.4, 44.4, 37.9, 37.6, 36.9, 36.8, 29.7, 25.2, 23.4, 23.2, 21.6, 18.5, 16.3. HRMS calculated mass: 425.0977 [M-H], measured: 425.0992 [M-H].

Compound **63**: NMR ^{1}H (300 MHz, $CDCl_3$) δ 7.25 (d, J = 8.4 Hz, 1H), 7.03 (d, J = 8.4 Hz, 1H), 3.42-3.30 (m, 1H), 2.99-2.66 (m, 2H), 2.30 (d, J = 12.9 Hz, 1H), 2.17 (d, J = 12.3Hz, 1H), 1.95- 1.40 (m, 7H), 1.29 (s, 3H), 1.25-1.16 (m, 9H). ^{13}C (75 MHz, $CDCl_3$) δ 184.5, 149.2, 148.6, 137.7, 124.7, 123.3, 111.3, 47.4, 44.1, 39.6, 38.8, 38.5, 37.5, 36.7, 25.3, 23.5, 23.2, 22.8, 18.8, 16.4. HRMS calculated mass: 425.0977 [M-H], measured: 425.0987 [M-H].

Synthesis of compound 42

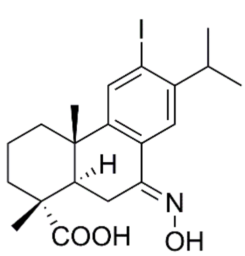

(1R,4aS,10aR)-9-(hydroxyimino)-6-iodo-7-isopropyl-1,4a-dimethyl-1,2,3,4,4a,9,10,10a-octahydrophenanthrene-1-carboxylic acid (**42**): Followed the general procedure B, ketone **43** (20.5 mg, 0.047 mMol), hydroxylamine hydrochloride (6.5 mg, 0.093 mMol) and pyridine (7.7 mg, 0.112 mMol) were used and compound **42** (20.5 mg, 97% yield) was achieved. NMR ^{1}H (500 MHz, $CDCl_3$) δ 7.70 (s, 1H), 7.52 (s, 1H), 3.08 (m, 1H), 2.83 (dd, J = 19.0, 5.5

Hz, 1H), 2.69 (dd, J = 19.0, 14.0 Hz, 1H), 2.31 (dd, J = 14.0, 5.5 Hz, 1H), 2.24 (br d, J = 12.5 Hz, 1H), 1.82-1.68 (m, 4H), 1.65-1.55 (m, 1H), 1.39 (s, 3H), 1.18-1.08 (m, 9H). ^{13}C (125 MHz, $CDCl_3$) δ 183.3, 155.1, 150.7, 148.6, 134.7, 129.0, 121.8, 104.0, 46.1, 41.5, 37.9, 37.3, 37.2, 36.6, 24.2, 23.2, 23.0, 22.9, 18.1, 16.6.

Synthesis of compound 43

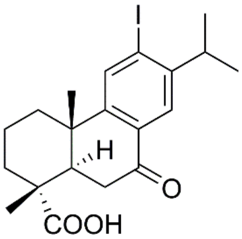

(1R,4aS,10aR)-6-iodo-7-isopropyl-9-oxo-1,4a-dimethyl-1,2,3,4,4a,9,10,10a-octahydrophenanthrene-1-carboxylic acid (**43**): Followed the general procedure A, compound **41** (300.0 mg, 0.704 mMol) and CrO_3 (98.5 mg, 0.985 mMol) were used as started materials, the mixture in HOAc was heated at 50°C for 3 h, then overnight at rt. Concentrated and purified on silica gel to give compound **43** (205.1 mg, yield 66%). NMR ^{1}H (300 MHz, $CDCl_3$) δ 7.822 (s, 1H), 7.817 (s, 1H), 3.15 (m, 1H), 2.80-2.60 (m, 2H), 2.48 (d, J = 14.1 Hz, 1H), 2.30 (d, J = 12.3 Hz, 1H), 1.85-1.70 (m, 4H), 1.70-1.55 (m, 1H), 1.33 (s, 3H), 1.30-1.18 (m, 9H). ^{13}C (75 MHz, $CDCl_3$) δ 198.4, 183.2, 154.0, 149.1, 135.3, 131.1, 124.6, 109.9, 46.5, 43.6, 38.0, 37.8, 37.3, 37.1, 36.6, 23.7, 23.2, 23.0, 18.1, 16.3. HRMS calculated mass: 441.0927 [M+H], measured: 441.0921 [M+H].

Synthesis of compound 44

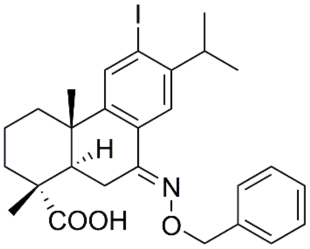

(1R,4aS,10aR)-9-((benzyloxy)imino)-6-iodo-7-isopropyl-1,4a-dimethyl-1,2,3,4,4a,9,10,10a-octahydrophenanthrene-1-carboxylic acid (**44**): Followed the general procedure B, ketone **43** (20.5 mg, 0.047 mMol), O-benzylhydroxylamine hydrochloride (16.3 mg, 0.093 mMol) and pyridine (7.7 mg, 0.112 mMol) were used and compound **44** (18.8 mg, 74% yield) NMR ^{1}H (500 MHz, $CDCl_3$) δ 7.74 (s, 1H), 7.67 (s, 1H), 7.41 (d, J = 7.0Hz, 2H), 7.38-7.27 (m, 3H), 5.23 (s, 2H), 3.11 (m, 1H), 2.74 (dd, J = 18.5, 5.0 Hz, 1H), 2.60 (dd, J = 19.0, 13.5 Hz, 1H), 2.28-2.18 (m, 2H), 1.80-1.68 (m, 4H), 1.65-1.55 (m, 1H), 1.34 (s, 3H), 1.25 (d, J = 7.0 Hz, 3H), 1.21 (d, J = 7.0 Hz, 3H), 1.09 (s, 3H). ^{13}C (125 MHz, $CDCl_3$) δ 182.8, 153.8, 150.1, 148.2, 138.2, 134.4, 129.8, 128.5, 128.4, 127.9, 122.0, 103.4, 76.6, 46.2, 41.5, 37.9, 37.3, 37.1, 36.4,

24.4, 23.3, 23.1, 23.0, 18.1, 16.5. HRMS calculated mass: 546.1505 [M+H], measured: 546.1500 [M+H].

Synthesis of compound 45

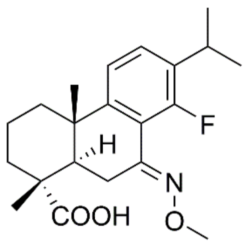

(1R,4aS,10aR)-8-fluoro-7-isopropyl-9-(methoxyimino)-1,4a-dimethyl-1,2,3,4,4a,9,10,10a-octahydrophenanthrene-1-carboxylic acid (**45**): Followed the general procedure B, compound **48** (8.8 mg, 0.026 mMol), O-methylhydroxylamine hydrochloride (4.4 mg, 0.053 mMol) and pyridine (4.4 mg, 0.055 mMol) were used and compound **45** (8.1 mg, 85% yield) was achieved. NMR ^{1}H (300 MHz, $CDCl_3$) δ 7.18 (dd, J = 8.1. 7.2 Hz), 6.99 (d, J = 8.4 Hz, 1H), 4.04 (s, 3H), 3.26 (m, 1H), 2.79 (dd, J = 18.6, 12.9 Hz, 1H), 2.62 (dd, J = 18.6, 5.4 Hz, 1H), 2.28-2.13 (m, 2H), 1.81-1.59 (m, 5H), 1.37 (s, 3H), 1.25 (d, J = 7.2 Hz, 3H), 1.21 (d, J = 7.2 Hz, 3H), 1.10 (s, 3H). ^{13}C (75 MHz, $CDCl_3$) δ 183.1, 157.6 (d, J_{CF} = 254.1 Hz), , 152.0 (d, J_{CF} = 6.8 Hz), 151.2, 134.1 (d, J_{CF} = 16.1 Hz), 127.4 (d, J_{CF} = 6.8 Hz), 118.1 (d, J_{CF} = 3.5 Hz), 117.5 (d, J_{CF} = 11.5 Hz), 62.4, 46.2, 41.2, 37.6, 37.3, 37.1, 26.8 (d, J = 4.7 Hz), 24.8, 23.2, 22.4, 22.3, 18.1, 16.7. ^{19}F (282.2 MHz, $CDCl_3$) δ -118.5 (d, J = 7.5 Hz). HRMS calculated mass: 362.2132 [M+H], measured: 362.2127 [M+H].

Synthesis of compound 46

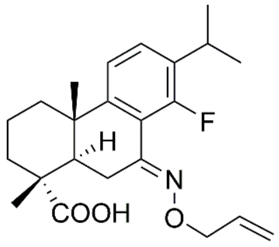

(1R,4aS,10aR)-9-((allyloxy)imino)-8-fluoro-7-isopropyl-1,4a-dimethyl-1,2,3,4,4a,9,10,10a-octahydrophenanthrene-1-carboxylic acid (**46**): Followed the general procedure B, compound **48** (11.0 mg, 0.033 mMol), O-allylhydroxylamine hydrochloride (7.6 mg, 0.069 mMol) and pyridine (5.7 mg, 0.073 mMol) were used and compound **46** (11.9 mg, 93% yield) was achieved. NMR ^{1}H (300 MHz, $CDCl_3$) δ 7.18 (dd, J = 8.1. 7.2 Hz, 1H), 6.99 (d, J = 8.1 Hz, 1H), 6.19-6.02 (m, 1H), 5.39-5.28 (m, 1H), 5.27-5.19 (m, 1H), 4.80-4.65 (m, 2H), 3.28 (m, 1H), 2.82 (dd, J = 19.2, 13.5 Hz, 1H), 2.65 (dd, J = 18.9, 6.0 Hz, 1H), 2.26-2.15 (m, 2H), 1.81-1.59 (m, 5H), 1.37 (s, 3H), 1.25 (d, J = 6.9 Hz, 3H), 1.21 (d, J = 6.9 Hz, 3H), 1.10 (s, 3H). ^{13}C (75 MHz, $CDCl_3$) δ 183.5, 157.6 (d, J_{CF} = 254.2 Hz) , 152.1 (d, J_{CF} = 6.9 Hz), 151.2, 134.8, 134.0 (d, J_{CF} = 16.0 Hz), 127.3 (d, J_{CF} = 6.8 Hz), 118.0 (d, J_{CF} = 3.5 Hz), 117.5 (d, J_{CF} = 13.7 Hz), 117.4, 75.5, 46.2, 41.2, 37.6, 37.4, 37.1, 26.8 (d, J = 3.5 Hz), 25.0, 23.3, 22.4, 22.3, 18.1, 16.7. ^{19}F (282.2 MHz, $CDCl_3$) δ -118.6 (d, J = 6.8 Hz). HRMS calculated mass: 388.2288 [M+H], measured: 388.2283 [M+H].

Synthesis of compound 47
Please find information together with compound **25**.

Synthesis of compound 48
Please find information together with compound **26**.

Synthesis of compound 49

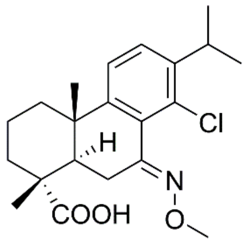

(1R,4aS,10aR)--8-chloro-7-isopropyl-9-methoxyimino-1,4a-dimethyl-1,2,3,4,4a,9,10,10a-octahydrophenanthrene-1-carboxylic acid (**49**): Followed the general procedure B, ketone **53** (24.1 mg, 0.069 mMol), O-methylhydroxylamine hydrochloride (11.5 mg, 0.138 mMol) and pyridine (11.5mg, 0.145 mMol) were used and compound **49** (12.6 mg, 48% yield) was achieved. NMR ^{1}H (300 MHz, $CDCl_3$) δ 7.22 (d, *J* = 8.1 Hz, 1H), 7.12 (d, *J* = 8.1 Hz, 1H), 4.04 (s, 3H), 3.54 (m, 1H), 3.01 (dd, *J* = 18.6, 12.6 Hz, 1H), 2.46 (dd, *J* = 18.6, 6.0 Hz, 1H), 2.21 (br d, *J* = 12.3 Hz, 1H), 2.13 (dd, *J* = 12.9, 6.6 Hz, 1H), 1.82-1.58 (m, 5H), 1.39 (s, 3H), 1.25 (d, *J* = 6.6 Hz, 3H), 1.19 (d, *J* = 6.9 Hz, 3H), 1.08 (s, 3H). ^{13}C (75 MHz, $CDCl_3$) δ 182.5, 152.9, 151.4, 145.4, 130.9, 128.5, 126.7, 121.0, 61.4, 46.0, 41.4, 37.8, 37.6, 37.2, 30.4, 24.9, 23.3, 22.6, 21.5, 18.0, 16.8. HRMS calculated mass: 378.1836 [M+H], 380.1806 [M+H+2], measured: 378.1832 [M+H], 380.1810 [M+H+2].

Synthesis of compound 50

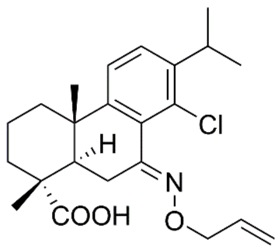

(1R,4aS,10aR)-9-((allyloxy)imino)-8-chloro-7-isopropyl-1,4a-dimethyl-1,2,3,4,4a,9,10,10a-octahydrophenanthrene-1-carboxylic acid (**50**): Followed the general procedure B, ketone **53** (21.7 mg, 0.062 mMol), O-allylhydroxylamine hydrochloride (13.6mg, 0.124 mMol) and pyridine (9.8 mg, 0.130 mMol) were used and compound **50** (12.5 mg, 50% yield) was achieved. NMR ^{1}H (300 MHz, $CDCl_3$) δ 7.22 (d, *J* = 8.7 Hz, 1H), 7.12 (d, *J* = 8.4 Hz, 1H), 6.20-6.02 (m, 1H), 5.35 (d, *J* = 16.5 Hz, 1H), 5.23 (d, *J* = 9.9 Hz, 1H), 4.80-4.65 (m, 2H), 3.53 (m, 1H), 3.05 (dd, *J* = 18.6, 12.9 Hz, 1H), 2.49 (dd, *J* = 18.6, 6.6 Hz, 1H), 2.20 (br d, *J* = 11.1 Hz, 1H), 2.13 (dd, *J* = 12.9, 6.6 Hz, 1H), 1.82-1.58 (m, 5H), 1.38 (s, 3H), 1.25 (d, *J* = 7.2 Hz, 3H), 1.19 (d, *J* = 6.6 Hz, 3H), 1.08 (s, 3H). ^{13}C (75 MHz, $CDCl_3$) δ 183.8, 153.1,

151.3, 145.4, 135.1, 130.9, 128.6, 126.7, 120.9, 117.4, 75.4, 46.1, 41.3, 37.7, 37.6, 37.3, 30.4, 25.0, 23.3, 22.6, 21.5, 18.1, 16.7. HRMS calculated mass: 404.1993 [M+H], 406.1967 [M+H+2], measured: 404.1987 [M+H], 406.1963 [M+H+2].

Synthesis of compound 51

Please find information together with compound **29**.

Synthesis of compound 52

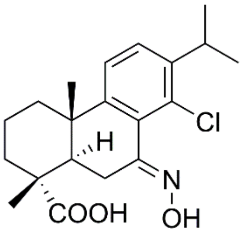

(1R,4aS,10aR)-8-chloro-9-(hydroxyimino)-7-isopropyl-1,4a-dimethyl-1,2,3,4,4a,9,10,10a-octahydrophenanthrene-1-carboxylic acid (**52**): Followed the general procedure B, ketone **53** (15.0 mg, 0.043 mMol), hydroxylamine hydrochloride (8.6 mg, 0.125 mMol) and pyridine (10.2 mg, 0.129 mMol) were used and product **52** (11.1 mg, 71% yield) was achieved. NMR ^{1}H (300 MHz, $CDCl_3$) δ 7.24 (d, J = 8.1 Hz, 1H), 7.14 (d, J = 8.1 Hz, 1H), 3.52 (m, 1H), 3.12 (dd, J = 18.6, 12.9 Hz, 1H), 2.55 (dd, J = 18.6, 6.0 Hz, 1H), 2.30-2.13 (m, 2H), 1.82-1.58 (m, 5H), 1.40 (s, 3H), 1.24 (d, J = 7.2 Hz, 3H), 1.19 (d, J = 7.2 Hz, 3H), 1.07 (s, 3H). ^{13}C (75 MHz, $CDCl_3$) δ 183.3, 154.4, 151.9, 145.5, 130.8, 127.7, 127.3, 121.3, 45.9, 41.1, 37.8, 37.2, 30.4, 25.2, 23.0, 22.7, 21.8, 18.1, 16.8.

Synthesis of compound 53

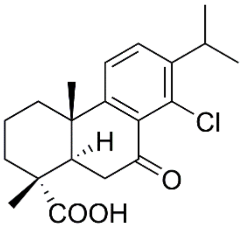

(1R,4aS,10aR)-8-chloro-7-isopropyl-9-oxo-1,4a-dimethyl-1,2,3,4,4a,9,10,10a-octahydrophenanthrene-1-carboxylic acid (**53**): Followed the general procedure A, compound **51** (107.0 mg, 0.320 mMol) and CrO_3 (38.3 mg, 0.383 mMol) were used as started material, the mixture was heated at 50°C for 3h, purified and compound **53** was got (80.0 mg, yield 72%). NMR ^{1}H (300 MHz, $CDCl_3$) δ 7.40 (d, J = 8.4 Hz, 1H), 7.22 (d, J = 8.4 Hz, 1H), 3.60-3.49 (m, 1H), 2.79-2.51 (m, 3H), 2.25 (d, J = 11.7 Hz, 1H), 1.85-1.55 (m, 5H), 1.34 (s, 3H), 1.24 (d, J = 7.2 Hz, 3H), 1.20 (d, J = 7.2 Hz, 3H), 1.17 (s, 3H). ^{13}C (75 MHz, $CDCl_3$) δ 197.7, 183.6, 154.6, 145.8, 132.2, 130.54, 130.46, 121.5, 45.9, 42.2, 39.0, 37.7, 37.5, 36.8, 29.7, 23.3, 22.9, 22.6, 18.1, 16.6. HRMS calculated mass: 349.1571 [M+H], 351.1541 [M+H+2], measured: 349.1566 [M+H], 351.1544 [M+H+2].

Synthesis of compound 54

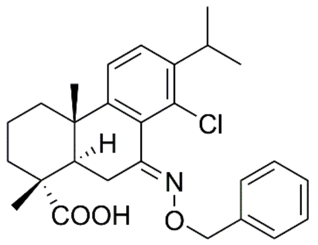

(1R,4aS,10aR)-9-((benzyloxy)imino)-8-chloro-7-isopropyl-1,4a-dimethyl-1,2,3,4,4a,9,10,10a-octahydrophenanthrene-1-carboxylic acid (**54**): Followed the general procedure B, ketone **53** (12.2 mg, 0.035 mMol), O-benzylhydroxylamine hydrochloride (11.2 mg, 0.070 mMol) and pyridine (5.8 mg, 0.074 mMol) were used and compound **54** (5.1 mg, 32% yield) was achieved. NMR ^{1}H (300 MHz, $CDCl_3$) δ 7.49-7.27 (m, 5H), 7.21 (d, J = 8.1 Hz, 1H), 7.11 (d, J = 8.1 Hz, 1H), 5.30 (d, J = 12.9 Hz, 1H), 5.24 (d, J = 12.9 Hz, 1H), 3.51 (m, 1H), 3.07 (dd, J = 18.6, 12.9 Hz, 1H), 2.49 (dd, J = 18.6, 6.3 Hz, 1H), 2.19 (br d, J = 11.7 Hz, 1H), 2.12 (dd, J = 12.9, 6.3 Hz, 1H), 1.80- 1.55 (m, 5H), 1.38 (s, 3H), 1.24 (d, J = 7.2 Hz, 3H), 1.19 (d, J = 7.2 Hz, 3H), 1.06 (s, 3H). ^{13}C (75 MHz, $CDCl_3$) δ 183.4, 153.4, 151.3, 145.3, 138.7, 131.0, 128.6, 128.43, 128.39, 127.8, 126.7, 120.9, 76.5, 46.0, 41.3, 37.7, 37.6, 37.3, 30.4, 25.2, 23.3, 22.6, 21.5, 18.1, 16.7. HRMS calculated mass: 454.2149 [M+H], 456.2120 [M+H+2], measured: 454.2143 [M+H], 456.2127 [M+H+2].

Synthesis of compound 55

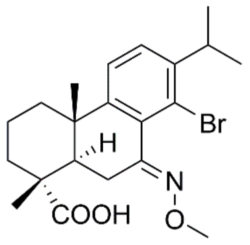

(1R,4aS,10aR)-8-bromo-7-isopropyl-9-(methoxyimino)-1,4a-dimethyl-1,2,3,4,4a,9,10,10a-octahydrophenanthrene-1-carboxylic acid (**55**): Followed the general procedure B, ketone **59** (20.0 mg, 0.051 mMol), O-methylhydroxylamine hydrochloride (8.5 mg, 0.102 mMol) and pyridine (8.9 mg, 0.112 mMol) were used and product **55** (17.0 mg, 79% yield) was achieved. NMR ^{1}H (500 MHz, $CDCl_3$) δ 7.21 (d, J = 8.5 Hz, 1H), 7.16 (d, J = 9.0 Hz, 1H), 4.05 (s, 3H), 3.57 (m, 1H), 3.05 (dd, J = 18.5, 13.0 Hz, 1H), 2.43 (dd, J = 18.5, 6.5 Hz, 1H), 2.21 (br d, J = 12.5 Hz, 1H), 2.13 (J = 13.0, 6.5 Hz, 1H), 1.81-1.70 (m, 4H), 1.65-1.55 (m, 1H), 1.39 (s, 3H), 1.25 (d, J = 7.0 Hz, 3H), 1.19 (d, J = 6.5 Hz, 3H), 1.08 (s, 3H). NMR ^{13}C (75 MHz, $CDCl_3$) δ 183.6, 153.7, 151.5, 147.3, 130.5, 127.0, 122.6, 121.7, 62.4, 46.1, 41.4, 37.8, 37.2, 33.4, 24.8, 23.5, 22.9, 21.4, 18.1, 16.7. HRMS calculated mass: 422.1331 [M+H], 424.1310 [M+H+2], measured: 422.1325 [M+H], 424.1308 [M+H+2].

Synthesis of compound 56

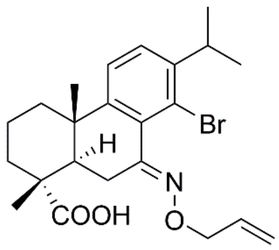

(1R,4aS,10aR)-9-((allyloxy)imino)-8-bromo-7-isopropyl-1,4a-dimethyl-1,2,3,4,4a,9,10,10a-octahydrophenanthrene-1-carboxylic acid (**56**): Followed the general procedure B, ketone **59** (20.2 mg, 0.051 mMol), O-allylhydroxylamine hydrochloride (11.3 mg, 0.103 mMol) and pyridine (8.5 mg, 0.107 mMol) were used and product **56** (18.4 mg, 80% yield) was achieved. NMR ^{1}H (300 MHz, $CDCl_3$) δ 7.21 (d, J = 8.1 Hz, 1H), 7.15 (d, J = 8.4 Hz, 1H), 6.20-6.05 (m, 1H), 5.35 (dd J = 17.1, 1.2 Hz, 1H), 5.23 (dd, J = 10.5, 1.2 Hz, 1H), 4.80-4.65 (m, 2H), 3.56 (m, 1H), 3.09 (dd, J = 18.0, 12.9 Hz, 1H), 2.46 (dd, J = 18.0, 6.6 Hz, 1H), 2.20 (br d, J = 11.1 Hz, 1H), 2.14 (dd, J = 12.9, 6.6 Hz, 1H), 1.82-1.57 (m, 5H), 1.39 (s, 3H), 1.25 (d, J = 6.6 Hz, 3H), 1.19 (d, J = 6.9 Hz, 3H), 1.08 (s, 3H). NMR ^{13}C (75 MHz, $CDCl_3$) δ 183.8, 153.9, 151.5, 147.2, 135.3, 130.6, 127.0, 122.6, 121.7, 117.4, 75.4, 46.1, 41.4, 37.7, 37.2, 33.4, 24.9, 23.5, 22.9, 22.8, 21.4, 18.1, 16.7. HRMS calculated mass: 448.1487 [M+H], 450.1467 [M+H+2], measured: 448.1482 [M+H], 450.1465 [M+H+2].

Synthesis of compound 57

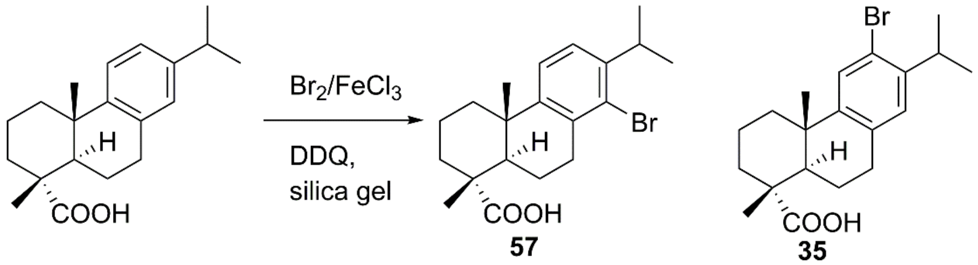

Supplemetary Scheme 7. Synthesis of compound **57.**
(1R,4aS,10aR)-8-bromo-7-isopropyl-1,4a-dimethyl-1,2,3,4,4a,9,10,10a-octahydrophenanthrene-1-carboxylic acid (**57**): To the mixture of $FeCl_3$ (3.6 mg, 0.022 mMol), DDQ (2.8 mg, 0.016 mMol), and Merk silica gel 60 (220.0 mg) in DCM added dehydroabietic acid (400.0 mg, 1.332 mMol), then 41,2 uL Br_2 (127.8 mg, 0.8 mMol) was added at 0°C and stirred at rt for about 40 min, about 25% conversed, then 82.4 uL Br_2 was added and stirred at rt, monitored with LC, full conversed was achieved after about 1 h at rt, concentrated and purified on silica gel with EtOAc/n-heptane/HCOOH (25:75:0.1 to 45:55:0.1) twice to give compound **57** (126.5 mg, yield 25%) and compound **35** (275.7 mg, yield 55%). Compound **57**: NMR ^{1}H (300 MHz, $CDCl_3$) δ 7.21 (d, J = 8.1 Hz, 1H), 7.09 (d, J = 8.4 Hz, 1H), 3.45 (m, 1H), 3.00 (dd, J = 18.0, 6.9 Hz, 1H), 2.90-2.74 (m, 1H), 2.31 (br d, J = 12.9 Hz, 1H), 2.18 (dd, 12.3, 1.8 Hz, 1H), 1.90-1.50 (m, 7H), 1.29 (s, 3H), 1.27-1.18 (m, 9H). NMR ^{13}C (75 MHz, $CDCl_3$) δ 185.0, 149.3, 145.0, 135.0, 128.0, 123.9, 123.5, 47.4, 43.9, 38.4, 37.4, 36.7, 33.1, 32.8, 25.2, 23.2, 22.9, 22.0, 18.7, 16.3.

Synthesis of compound 58

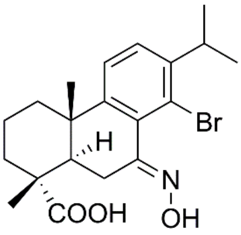

(1R,4aS,10aR)-8-bromo-9-(hydroxyimino)-7-isopropyl-1,4a-dimethyl-1,2,3,4,4a,9,10,10a-octahydrophenanthrene-1-carboxylic acid (**58**): Followed the general procedure B, ketone **59** (9.5 mg, 0.024 mMol), hydroxylamine hydrochloride (5.0 mg, 0.073 mMol) and pyridine (5.9 mg, 0.075 mMol) were used and product **58** (6.0 mg, 61% yield) was achieved. NMR ^{1}H (500 MHz, $CDCl_3$) δ 7.23 (d, J = 8.0 Hz, 1H), 7.17 (d, J = 8.5 Hz, 1H), 3.54 (m, 1H), 3.17 (dd, J = 18.5, 13.0 Hz, 1H), 2.53 (dd, J = 18.5, 7.0 Hz, 1H), 2.24-2.15 (m, 2H), 1.82-1.70 (m, 4H), 1.68-1.58 (m, 1H), 1.40 (s, 3H), 1.24 (d, J = 7.0 Hz, 3H), 1.20 (d, J = 6.5 Hz, 3H), 1.07 (s, 3H). ^{13}C (125 MHz, $CDCl_3$) δ 182.8, 155.1, 151.8, 147.2, 130.5, 127.2, 122.3, 121.8, 46.0, 41.5, 37.85, 37.80, 37.3, 33.4, 24.5, 23.3, 23.0, 21.5, 18.1, 16.8.

Synthesis of compound 59

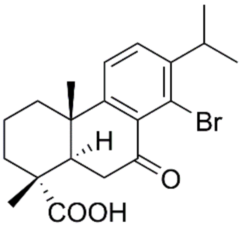

(1R,4aS,10aR)-8-bromo-7-isopropyl-9-oxo-1,4a-dimethyl-1,2,3,4,4a,9,10,10a-octahydrophenanthrene-1-carboxylic acid (**59**): Followed the general procedure A, compound **57** (126.5 mg, 0.334 mMol) and CrO_3 (65.1 mg, 0.651 mMol) were used as started materials, the mixture was heated at 50°C for 5.5 h, purified and compound **59** was got (77.0 mg, yield 59%). NMR ^{1}H (500 MHz, $CDCl_3$) δ 7.38 (d, J = 8.5 Hz, 1H), 7.26 (d, J = 8.5 Hz, 1H), 3.60 (m, 1H), 2.80-2.59 (m, 3H), 2.26 (d, J = 13.0 Hz, 1H), 1.83-1.70 (m, 4H), 1.70-1.60 (m, 1H), 1.36 (s, 3H), 1.34-1.15 (m, 9H). NMR ^{13}C (125 MHz, $CDCl_3$) δ 198.3, 183.6, 154.7, 147.6, 132.5, 130.5, 122.8, 122.2, 45.9, 42.1, 38.8, 37.7, 37.5, 36.8, 32.6, 23.2, 23.1, 22.8, 18.1, 16.6. HRMS calculated mass: 393.1065 [M+H], 395.1045 [M+H+2], measured: 393.1060 [M+H], 395.1043 [M+H+2].

Synthesis of compound 60

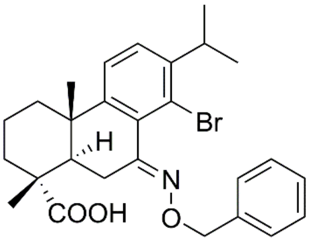

(1R,4aS,10aR)-9-((benzyloxy)imino)-8-bromo-7-isopropyl-1,4a-dimethyl-1,2,3,4,4a,9,10,10a-octahydrophenanthrene-1-carboxylic acid (**60**): Followed the general procedure B, ketone **59** (11.0 mg, 0.028 mMol), O-benzylhydroxylamine hydrochloride (9.7mg, 0.056 mMol) and pyridine (4.7 mg, 0.059 mMol) were used and product **60** (10.0 mg, 72% yield) was achieved. NMR ^{1}H (300 MHz, $CDCl_3$) δ 7.45 (d, *J* = 6.9 Hz, 2H), 7.40-7.25 (m, 3H), 7.20 (d, *J* = 8.1 Hz, 1H), 7.14 (d, *J* = 8.4 Hz, 1H), 5.28 (s, 2H), 3.53 (m, 1H), 3.13 (dd, *J* = 18.9, 12.9, 1H), 2.48 (dd, *J* = 18.9, 6.6 Hz, 1H), 2.19 (br d, *J* = 11.7 Hz, 1H), 2.13 (dd, *J* = 12.9, 6.3 Hz, 1H), 1.80-1.52 (m, 5H), 1.38 (s, 3H), 1.24 (d, *J* = 6.9 Hz, 3H), 1.17 (d, *J* = 6.3 Hz, 3H), 1.06 (s, 3H). ^{13}C (75 MHz, CDCl3) δ 183.4, 154.2, 151.5, 147.2, 138.8, 130.7, 128.43, 128.39, 127.8, 127.0, 122.6, 121.6, 76.5, 46.0, 41.4, 37.7, 37.3, 33.4, 25.0, 23.5, 22.8, 21.4, 18.1, 16.7. HRMS calculated mass: 498.1644 [M+H], 500.1623 [M+H+2], measured: 498.1638 [M+H], 500.1622 [M+H+2].

Synthesis of compound 61

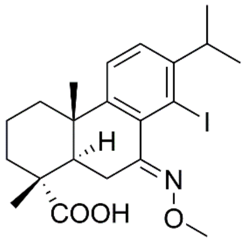

(1R,4aS,10aR)-)-8-iodo-7-isopropyl-9-methoxyimino-1,4a-dimethyl-1,2,3,4,4a,9,10,10a-octahydrophenanthrene-1-carboxylic acid (**61**): Followed the general procedure B, ketone **64** (8.8 mg, 0.020 mMol), O-methylhydroxylamine hydrochloride (3.3 mg, 0.040 mMol) and pyridine (3.3 mg, 0.042 mMol) were used and product **61** (4.5 mg, 51% yield) was achieved. NMR ^{1}H (300 MHz, $CDCl_3$) δ 7.19 (d, *J* = 8.1 Hz, 1H), 7.15 (d, *J* = 8.1 Hz, 1H), 4.07 (s, 3H), 3.45 (m, 1H), 3.08 (dd, *J* = 18.3, 12.9 Hz, 1H), 2.42 (dd, *J* = 18.3, 6.3 Hz, 1H), 2.21 (br d, *J* = 12.3 Hz, 1H), 2.14 (dd, *J* = 12.9, 6.3 Hz, 1H), 1.80-1.53 (m, 5H), 1.39 (s, 3H), 1.24 (d, *J* = 6.3 Hz, 3H), 1.19 (d, *J* = 7.2 Hz, 3H), 1.07 (s, 3H). ^{13}C (75 MHz, $CDCl_3$) δ 182.3, 155.2, 150.9, 150.7, 134.0, 126.7, 122.7, 101.5, 62.3 46.0, 41.5, 39.3, 37.70, 37.65, 37.1, 24.5, 23.8, 23.2, 21.3, 18.0, 16.7. HRMS calculated mass: 470.1192 [M+H], measured: 470.1186 [M+H].

Synthesis of compound 62

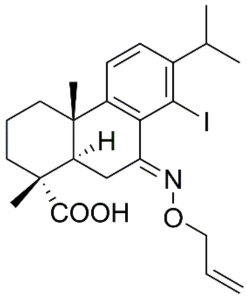

(1R,4aS,10aR)-9-((allyloxy)imino)-8-iodo-7-isopropyl-1,4a-dimethyl-1,2,3,4,4a,9,10,10a-octahydrophenanthrene-1-carboxylic acid (**62**): Followed the general procedure B, ketone **64** (6.0 mg, 0.014 mMol), O-Allylhydroxylamine hydrochloride (4.5 mg, 0.041 mMol) and pyridine (3.3 mg, 0.042 mMol) were used and compound **62** (5.9 mg, 88% yield) was achieved. NMR ^{1}H (300 MHz, $CDCl_3$) δ 7.19 (d, J = 8.1 Hz, 1H), 7.15 (d, J = 8.1 Hz, 1H), 6.25-6.08 (m, 1H), 5.36 (dd, J = 17.1, 1.8 Hz, 1H), 5.24 (dd, J = 10.5, 1.8 Hz, 1H), 4.82-4.68 (m, 2H), 3.47 (m, 1H), 3.13 (dd, J = 18.0, 12.9 Hz, 1H), 2.41 (dd, J = 18.0, 6.3 Hz, 1H), 2.25-2.09 (m, 2H), 1.82-1.70 (m, 4H), 1.66-1.57 (m, 1H), 1.39 (s, 3H), 1.24 (d, J = 7.2 Hz, 3H), 1.18 (d, J = 6.9 Hz, 3H), 1.07 (s, 3H). ^{13}C (75 MHz, $CDCl_3$) δ 182.9, 155.6, 150.9, 150.7, 135.4, 134.2, 126.6, 122.6, 117.5, 101.4, 75.4, 46.0, 41.5, 39.3, 37.7, 37.2, 24.7, 23.8, 23.2, 21.4, 18.0, 16.7. HRMS calculated mass: 496.1349 [M+H], measured: 496.1342 [M+H].

Synthesis of compound 63

Please find information together with compound **41**.

Synthesis of compound 64

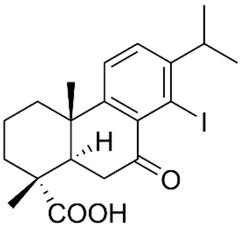

(1R,4aS,10aR)-8-iodo-7-isopropyl-1,4a-dimethyl-9-oxo-1,2,3,4,4a,9,10,10a-octahydrophenanthrene-1-carboxylic acid (**64**): Followed the general procedure A, compound **63** (48.2 mg, 0.113 mMol) and CrO_3 (15.8 mg, 0.158 mMol) were used as started materials, then the mixture in HOAc was heated at 50°C for 3 h, then overnight at rt. Concentrated and purified on silica gel to give compound **64** (6.4 mg, yield 13%). NMR ^{1}H (300 MHz, $CDCl_3$) δ 7.32 (d, J = 8.1 Hz, 1H), 7.25 (d, J = 8.1 Hz, 1H), 3.54 (m, 1H), 2.82-2.55 (m, 3H), 2.25 (d, J = 11.7 Hz, 1H), 1.82-1.56 (m, 5H), 1.35 (s, 3H), 1.23 (d, J = 6.6 Hz, 3H), 1.19 (d, J = 6.9 Hz, 3H), 1.16 (s, 3H). ^{13}C (75 MHz, $CDCl_3$) δ 199.3, 183.0, 154.4, 151.1, 135.4, 129.9, 123.2, 100.2, 45.8, 42.0, 38.3, 37.7, 37.5, 36.8, 23.4, 23.2, 23.1, 18.1, 16.6. HRMS calculated mass: 441.0927 [M+H], measured: 441.0922 [M+H].

Synthesis of compounds 73 and 77:

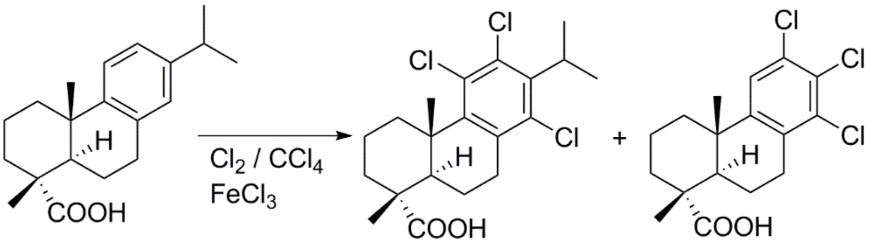

73 **77**

Supplementary Scheme 5. Synthesis of compounds **73** and **77**
(1R,4aS,10aR)-5,6,8-trichloro-7-isopropyl-1,4a-dimethyl-1,2,3,4,4a,9,10,10a-octahydrophenanthrene-1-carboxylic acid (**73**), (1R,4aS,10aR)-6,7,8-trichloro-1,4a-dimethyl-1,2,3,4,4a,9,10,10a-octahydrophenanthrene-1-carboxylic acid (**77**): To the mixture of dehydroabietic acid (200 mg, 0.666 mMol), $FeCl_3$ (49.7 mg, 0.306 mMol), DDQ (2 mg, 0.0088 mMol) and silica gel (12 mg) added 10 mL Cl_2 in CCl_4 at 0ºC and stirred at this temperature for about 2.5 h, The temperature was allowed to warm up rt and stirred overnight. The excess amount of Cl_2 was flushed away with air and concentrated, purified on silica gel with EtOAc/n-heptane/HCOOH (25:75:0.1 to 50:50:0.1) to give compound **73** (153.0 mg, 57% yield), and 50.5 mg mixture, which was purified further with preparative HPLC to give compound **77** (16.2 mg, 7%). Compound **73**: NMR ^{1}H (300 MHz, $CDCl_3$) δ 4.03 (m, 1H), 3.55-3.41 (m, 1H), 2.90-2.80 (m, 2H), 2.09 (d, J = 12.0 Hz, 1H), 1.80-1.56 (m, 6H), 1.51 (s, 3H), 1.41 (d, J = 6.0 Hz, 6H), 1.32 (s, 3H), 1.28-1.15 (m, 1H).
Compound **77**: NMR ^{1}H (300 MHz, $CDCl_3$) δ 7.29 (s, 1H), 2.95 (dd, J = 18.0, 6.6 Hz, 1H), 2.95-2.70 (m, 1H), 2.25 (br d, J = 12.9 Hz, 1H), 2.13 (dd, J = 12.3 Hz, 1H), 1.90-1.60 (m, 6H), 1.55-1.39 (m, 1H), 1.28 (s, 3H), 1.21 (s, 3H). ^{13}C (75 MHz, $CDCl_3$) δ 184.4 (COOH), 150.4 (C4b), 134.2 (C8a), 134.0 (C8), 131.0 (C7), 128.7 (C6), 124.6 (C5), 47.3 (C1), 43.6 (C10a), 38.1 (C2), 37.5 (C4a), 36.6 (C4), 29.3 (C9), 25.1 (4a-Me), 21.2 (C10), 18.5 (C3), 16.4 (1-Me). HRMS calculated mass: 359.0372 [M-H], 361.0343 [M-H+2], 363.0313 [M-H+4], measured: 359.0378 [M-H], 361.0350 [M-H+2]. 363.0321 [M-H+4].

Synthesis of compound 75

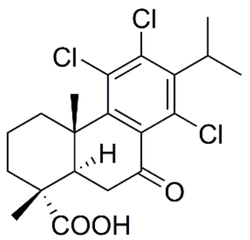

(1R,4aS,10aR)-5,6,8-trichloro-7-isopropyl-1,4a-dimethyl-9-oxo-1,2,3,4,4a,9,10,10a-octahydrophenanthrene-1-carboxylic acid (**75**): Followed the general procedure A, 11,12,14-trichlorodehydroabietic acid (128.0 mg, 0.317 mMol) and CrO_3 (44.4 mg, 0.444 mMol) were used as started material, the mixture was heated at 50°C for 2.5 h, then at rt overnight, purified and compound **75** (62.0 mg, yield 47%) was got. NMR ^{1}H (300 MHz, $CDCl_3$) δ 4.07 (m, 1H), 3.48 (d, J = 13.5 Hz, 1H), 2.75-2.54 (m, 3H), 1.85-1.70 (m, 5H), 1.42 (s, 3H), 1.41 (d, J = 6.0 Hz, 6H), 1.34 (s, 3H). ^{13}C (75 MHz, $CDCl_3$) δ 196.6, 150.6, 144.1, 139.6, 132.8, 46.7, 43.3, 42.5, 37.9, 36.3, 35.7, 19.1, 18.2, 17.3, 16.7. HRMS calculated mass: 417.0791 [M+H], 419.0762 [M+H+2], 421.0732 [M+H+4], measured: 417.0784 [M+H], 419.0758 [M+H+2]. 421.0730 [M+H+4].

Synthesis of compound 76

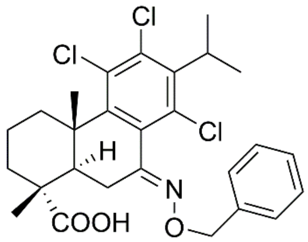

(1R,4aS,10aR)-9-((benzyloxy)imino)-5,6,8-trichloro-7-isopropyl-1,4a-dimethyl-1,2,3,4,4a,9,10,10a-octahydrophenanthrene-1-carboxylic acid (**76**): Followed the general procedure B, ketone **75** (20.5 mg, 0.049 mMol), O-benzylhydroxylamine hydrochloride (15.7 mg, 0.098 mMol) and pyridine (8.2 mg, 0.103 mMol) were used and compound **76** (23.1 mg, 90% yield) was achieved. NMR ^{1}H (300 MHz, $CDCl_3$) δ 7.43-7.28 (m, 5H), 5.23 (d, J = 12.0 Hz, 1H), 5.18 (d, J = 12.0 Hz, 1H), 4.04 (m, 1H), 3.41 (br d, J = 15.0 Hz, 1H), 3.01 (dd, J = 18.0, 15.0 Hz, 1H), 2.46 (dd, J = 18.0, 6.0 Hz, 1H), 2.18 (dd, J = 12.0, 6.0 Hz, 1H), 1.75-1.62 (m, 5H), 1.40 (br d, J = 6.0 Hz, 6H), 1.35 (s, 3H), 1.24 (s, 3H). ^{13}C (75 MHz, $CDCl_3$) δ 183.4, 149.0, 143.3, 138.3, 135.8, 131.4, 128.5, 128.4, 128.0, 76.7, 47.0, 42.9, 42.4, 36.84, 36.76, 25.2, 19.4, 19.3, 18.3, 17.3, 16.8. HRMS calculated mass: 522.1370 [M+H], 524.1340 [M+H+2], 526.1311 [M+H+4], measured: 522.1363 [M+H], 524.1338 [M+H+2]. 526.1316 [M+H+4].

Synthesis of compound 77
Please find information together with compound **73**.

Synthesis of compound 78-79
Please find information together with compound **15**.

Synthesis of compound 80-81
Please find information together with compound **22**.

Synthesis of compound 82

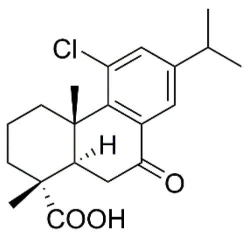

(1R,4aS,10aR)-5-chloro-7-isopropyl-1,4a-dimethyl-9-oxo-1,2,3,4,4a,9,10,10a-octahydrophenanthrene-1-carboxylic acid (**82**). Followed the general procedure A, compound **22** (20.9 mg, 0.062 mMol) and CrO_3 (9.4 mg, 0.094 mMol) were used as started materials, and then the mixture in HOAc was stirred at rt overnight. Concentrated and purified on silica gel with 25-45% EtOAc/n-heptane/HCOOH (25:75:0.1 to 45:55:0.1) to give compound **82**

(16.9 mg, yield 55%). NMR ^{1}H (300 MHz, $CDCl_3$) δ 7.92 (d, J = 2.1 Hz, 1H), 7.39 (d, J = 2.1 Hz, 1H), 3.55 (br d, J = 12.9 Hz, 1H), 2.87 (m, 1H), 2.76-2.70 (m, 2H), 2.44-2.30 (m, 1H), 1.88-1.70 (m, 5H), 1.52 (s, 3H), 1.36 (s, 3H), 1.24 (d, J = 6.9 Hz, 6H). ^{13}C (75 MHz, $CDCl_3$) δ 197.4, 181.8, 148.4, 147.1, 136.2, 134.2, 132.4, 125.3, 47.3, 44.4, 40.9, 37.6, 36.3, 35.2, 33.3, 23.63, 23.58, 20.5, 18.3, 17.6, 16.6.

References:

1. Cui, Y.M. *et al.* Design, synthesis, and characterization of BK channel openers based on oximation of abietane diterpene derivatives. *Bioorg. Med. Chem.*, **18** (24), 8642-8659 (2010)
2. Cui, Y.M. *et al.* Novel oxime and oxime ether derivatives of 12,14-dichlorodehydroabietic acid: Design, synthesis, and BK channel-opening activity. *Bioorg. Med. Chem. Lett.*, **18** (24), 6386-6389 (2008).
3. Ohwada, T. *et al*. Dehydroabietic acid derivatives as a novel scaffold for large-conductance calcium-activated K^+ channel openers. *Bioorg. Med. Chem. Lett.*, **13** (22), 3971-3974 (2003).
4. Dimitriadis Kutney, J.P. & Dimitriadis, E. Studies related to biological detoxification of kraft pulp mill effluent. V. The synthesis of 12- and 14-chlorodehydroabietic acids and 12,14-dichlorodehydroabietic acid, fish-toxic diterpenes from kraft pulp mill effluent. *Helv. Chim. Acta*, **65** (5), 1351 – 1358 (1982).
5. Wu, L. *et al.* Systematic phytochemical investigation of Abies spectabilis. *Chem. Pharm. Bull.*, **58** (12), 1646-1649 (2010).
